# Realization of strongly correlated 2D honeycomb boron

**DOI:** 10.1126/sciadv.aee3116

**Published:** 2026-07-01

**Authors:** Takemi Kato, Tomonori Nakamura, Kosuke Nakayama, Takumi Osumi, Seigo Souma, Asuka Honma, Alexandre Antezak, Pedro Rezende Gonçalves, Kiyohisa Tanaka, Miho Kitamura, Kenichi Ozawa, Koji Horiba, Hiroshi Kumigashira, Takashi Takahashi, Franck Fortuna, Andrés Felipe Santander-Syro, Rikio Settai, Yoshichika Onuki, Yoshinori Okada, Takafumi Sato

**Affiliations:** 1 Advanced Institute for Materials Research (WPI-AIMR), Tohoku University, Sendai 980-8577, Japan.; 2 Okinawa Institute of Science and Technology (OIST), Okinawa 904-0495, Japan.; 3 Department of Physics, Tohoku University, Sendai 980-8578, Japan.; 4 Center for Science and Innovation in Spintronics (CSIS), Tohoku University, Sendai 980-8577, Japan.; 5 Université Paris-Saclay, CNRS, Institut des Sciences Moléculaires d’Orsay (ISMO), 91405 Orsay, France.; 6 UVSOR Synchrotron Facility, Institute for Molecular Science, Okazaki 444-8585, Japan.; 7 School of Physical Sciences, The Graduate University for Advanced Studies (SOKENDAI), Okazaki 444-8585, Japan.; 8 National Institutes for Quantum Science and Technology (QST), Sendai 980-8579, Japan.; 9 Institute of Materials Structure Science, High Energy Accelerator Research Organization (KEK), Tsukuba 305-0801, Japan.; 10 Institute of Multidisciplinary Research for Advanced Materials (IMRAM), Tohoku University, Sendai 980-8577, Japan.; 11 Department of Physics, Niigata University, Niigata 950-2181, Japan.; 12 RIKEN Center for Emergent Matter Science (CEMS), Wako 351-0198, Japan.; 13 International Center for Synchrotron Radiation Innovation Smart (SRIS), Tohoku University, Sendai 980-8577, Japan.; 14 Mathematical Science Center for Co-creative Society (MathCCS), Tohoku University, Sendai 980-8578, Japan.

## Abstract

Borophene, a two-dimensional (2D) boron sheet, has recently gained considerable attention as a postgraphene material owing to its unique properties, such as intrinsic metallic character, lightest element monolayer structure, and high-frequency phonons. These features, together with its theoretically predicted high density of states, make borophene an excellent platform for investigating various quantum phenomena. However, the realization of emergent phenomena is hindered by fabrication challenges and inherently weak electronic correlations within the *s* and *p* electron system. Here, we demonstrate a strongly correlated 2D electronic state derived from a honeycomb boron lattice exposed at the surface of ternary boride LaRh_3_B_2_. Using angle-resolved photoemission spectroscopy, we uncover the extended saddle-point van Hove singularity near the Fermi level, which is linked to the substantial lattice expansion and many-body interaction. Moreover, scanning tunneling microscopy reveals the electronic nematicity, which likely originates from the saddle-point instability. Our findings open a pathway to exploring the exotic correlated phenomena in boron-based 2D systems.

## Teaser

Electronic nematicity emerges in a highly stretched honeycomb boron lattice stabilized at a bulk crystal surface.

## INTRODUCTION

**Fig. 1.**
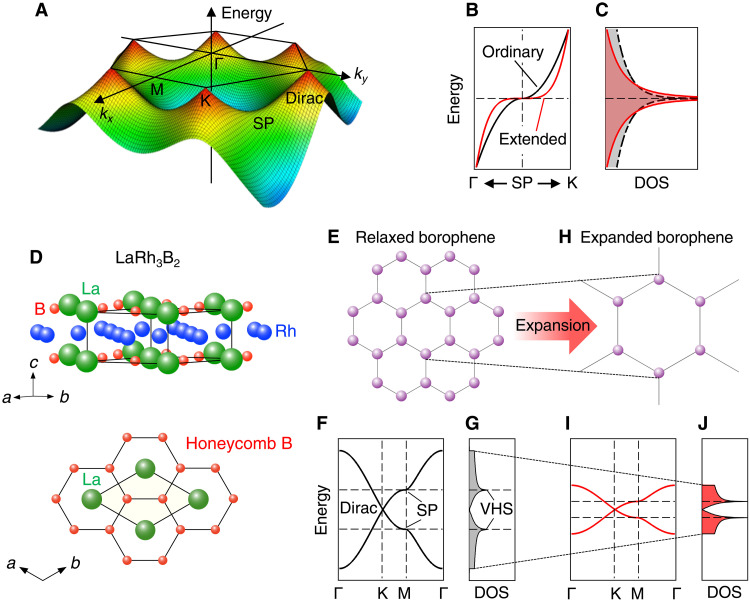
SPs and van Hove singularities in a honeycomb lattice. (**A**) Schematic SP band structure in a 2D momentum space (*E*, *k_x_*, and *k_y_*). (**B**) Band dispersion of ordinary (black) and extended (red) SPs along the orthogonal direction. (**C**) DOS highlighting the distinct VHS divergence for the ordinary and extended SPs. (**D**) Crystal structure of LaRh_3_B_2_, featuring a honeycomb boron lattice. (**E** to **G**) Honeycomb boron sheet (borophene), its band dispersion, and DOS calculated via a nearest-neighbor tight-binding model, respectively. (**H** to **J**) The same as (E) to (G) but for an expanded borophene sheet with a larger in-plane lattice constant.

Graphene has unveiled a distinct paradigm in materials science, characterized by its unique electronic structure, high carrier mobility, and remarkable mechanical flexibility (*1*–*3*). These properties arise from its two-dimensional (2D) honeycomb lattice, which gives rise to the peculiar electronic state (Fig. 1A) with massless Dirac fermions and exceptional conductivity. Beyond these notable characteristics, a long-standing challenge has been to induce strong correlation effects in graphene (*4*), which could lead to symmetry-breaking or topologically nontrivial phases. However, the intrinsic weakness of Coulomb interactions in this *s*- and *p*-electrons system has hindered the realization of such correlated phenomena. Conventional tuning methods, such as gating or doping (*3*), fail to sufficiently enhance electronic correlation and thus have not led to observable phase transition driven by many-body effects.

A notable breakthrough in overcoming this limitation has been achieved in twisted bilayer graphene, where moiré-induced flat minibands near the Fermi level (*E*_F_) provide a fertile ground for superconducting and correlated states (*5*–*7*). These phases emerge from a high density of states (DOS) even in an *s*/*p*-orbital system. However, the requirement of precise control over the twist angle and carrier density presents major challenges in terms of reproducibility and uniformity (*8*). An alternative strategy focuses on enhancing a correlation effect within the honeycomb lattice leveraging the saddle point (SP) at the M point in the Brillouin zone (Fig. 1A). These SPs, originating from the opposite curvatures of band dispersions (Fig. 1B), generate a van Hove singularity (VHS) that leads to a logarithmic DOS divergence in 2D (Fig. 1C) (*9*). Moreover, if the SP is extended by higher-order terms, such as quartic dispersion (*10*), the effective mass of SP band is renormalized (Fig. 1B), broadening the VHS region and further amplifies correlation effects (Fig. 1C). This mechanism offers a symmetry-protected and moiré-free pathway toward correlated physics. Nevertheless, experimental realization of extended SPs remains scarce (*11*), and a systematic approach to identifying such systems has yet to be established. While theoretical predictions of SP-induced correlated phenomena exist for doped graphene (*12*–*14*), their experimental verification remains an outstanding challenge.

To realize the SP physics and induce correlated electronic phases, a promising route is to reduce the electron filling within the honeycomb lattice. One feasible strategy is the substitution of carbon with boron, an element having one fewer valence electron. The resulting monolayer boron sheet—borophene—has recently attracted considerable attention as a postgraphene material. However, the synthesis of borophene remains experimentally challenging. The inherent electron deficiency of boron leads to a rich polymorphism with numerous possible allotropes, and stabilizing a pure honeycomb structure through conventional deposition is particularly difficult (*15*). To overcome the challenge in synthesis and achieve a honeycomb boron structure, here we propose to focus on the 2D lattice that naturally exists within stable bulk phases, providing a feasible pathway for the controlled synthesis of borophene-based electronic states.

In this study, we focus on ternary compounds *RT*_3_*X*_2_ (*R*: rare earth, *T*: transition metal, and *X*: B, Si, and Ga), which consist of a triangular lattice of *R*, a kagome lattice of *T*, and a honeycomb lattice of *X* embedded in the *R* plane (Fig. 1D) (*16*–*18*). Among the family, LaRh_3_B_2_, which is a rare superconducting example, crystallizes in the CoRh_3_B_2_-type structure with the space group *P*6/*mmm* (no. 191), preserving the perfect honeycomb lattice of boron atoms (Fig. 1D, bottom) (*16*). This honeycomb boron lattice (Fig. 1E) is generally expected to host a Dirac cone and SPs in the band structure (Fig. 1F), with the SPs giving rise to VHSs in DOS (Fig. 1G). Moreover, crucially, the honeycomb boron lattice in LaRh_3_B_2_ has a key structural advantage over known graphene systems: Notably, it is highly expanded (Fig. 1H) owing to the insertion of a lanthanum atom in the center of each boron hexagon (Fig. 1D, bottom). This unique structural property provides an excellent opportunity to explore the SP with a substantially reduced bandwidth and sharper DOS peaks (Fig. 1, I and J), which potentially drive the system toward correlation-induced instability when compared to borophene fabricated on metals.

Here, we demonstrate such a strongly renormalized 2D SP band at the cleaved surface of LaRh_3_B_2_ by angle-resolved photoemission spectroscopy (ARPES). First-principles band calculations, assuming a highly expanded borophene sheet, reproduce this SP band reasonably well. Furthermore, our scanning tunneling microscopy (STM) measurements reveal the rotational symmetry breaking, which could be induced by the electronic instability associated with the extended SP-VHS. The present results establish a promising material platform and strategy to access exotic electronic correlated phases near extended SPs.

## RESULTS

### Observation of 2D SP band structure in LaRh_3_B_2_

**Fig. 2.**
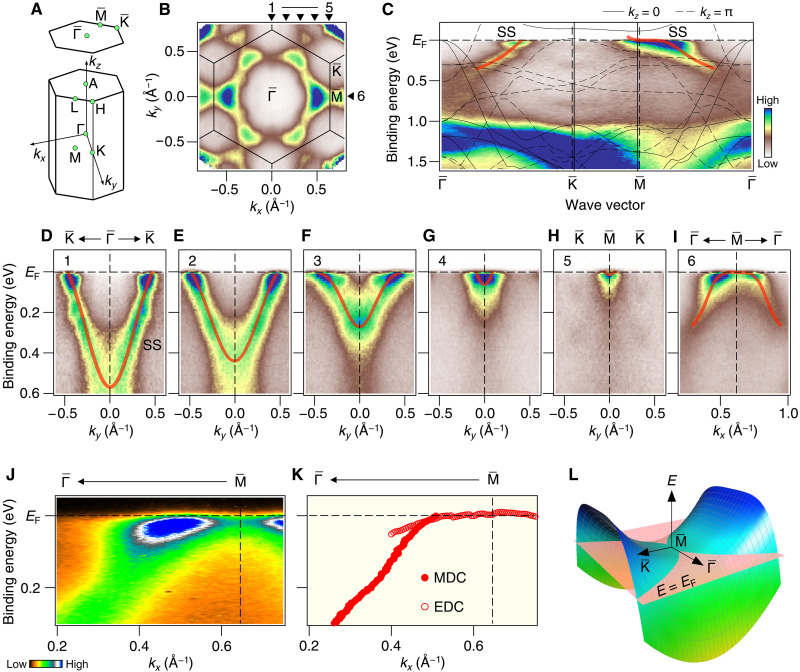
SP band structure in LaRh**_3_B_2_**. (**A**) Bulk and surface Brillouin zone of LaRh_3_B_2_. (**B**) ARPES-intensity map at *E*_F_ plotted as a function of *k_x_* and *k_y_* measured with *h*ν = 60 eV. (**C**) Corresponding valence-band structure along the high-symmetry axis. Solid (dashed) curves in (C) are calculated band dispersion for *k_z_* = 0 (*k_z_* = π) for bulk LaRh_3_B_2_. (**D** to **I**) Near-*E*_F_ ARPES intensity along cuts 1 to 6 indicated in (B). Red curves trace the dispersion of the SS band that forms an SP at the $\bar{M}$ point. (**J**) High-resolution near-*E*_F_ ARPES intensity around the $\bar{M}$ point. (**K**) Band dispersion of the SS band estimated by numerical fitting to momentum- and energy-distribution curves (MDCs and EDCs). (**L**) A schematic of the experimental band dispersion of the SS band around the $\bar{M}$ point.

First, we show in Fig. 2B the Fermi-surface mapping covering the first Brillouin zone projected on the surface (see Fig. 2A) measured at photon energy (*h*ν) of 60 eV. The most prominent feature is a distorted hexagonal contour centered at the $\bar{\Gamma }$ point, with vertices of strong intensity extending toward the $\bar{M}$ points, which is distinct from results reported in a previous study (see text S1) (*19*). The corresponding valence-band structure along the high-symmetry lines (Fig. 2C) indicates that, while the band forming the Fermi surface disperses within 0.5 eV from *E*_F_ along $\bar{\text{ΓK}}$ and $\bar{\text{ΓM}}$, it becomes flatter as it approaches the $\bar{M}$ point (see red curves). The extended high intensity around the $\bar{M}$ point in the Fermi-surface map arises from this flattening. In addition to the near-*E*_F_ band, several more dispersive bands appear at higher binding energies (*E*_B_s), including a hole-like dispersion topped at the $\bar{\Gamma }$ point. These high-*E*_B_ bands, particularly the $\bar{\Gamma }$-centered hole-like dispersion, are reasonably reproduced by bulk-band calculations (black curves) and attributed to the Rh-4*d* bands. By contrast, the near-*E*_F_ band is not captured by the bulk-band calculation, suggesting its surface origin. The *h*ν (*k_z_*) independence of this band, unlike the bulk bands showing *k_z_* dispersion, confirms its 2D character (see text S2). Henceforth, we refer to this 2D surface-derived state as the “SS band.”

To investigate the SS band dispersion in detail, we performed ARPES measurements near *E*_F_ along several *k* cuts (1 to 5) with different *k_x_* values, as illustrated by triangles in Fig. 2B. In cut 1 ($\bar{\text{ΓK}}$ cut; *k_x_* = 0) (Fig. 2D), the SS band exhibits an electron-like dispersion centered at the $\bar{\Gamma }$ point. With increasing *k_x_*, the band bottom progressively shifts upward (see cuts 2 to 4 in Fig. 2, E to G), and finally approaches *E*_F_ at cut 5 ($\bar{\text{MK}}$ cut), all the while retaining its electron-like dispersion. In contrast, along the orthogonal cut 6 ($\bar{\mathrm{M\Gamma}}$ cut; Fig. 2I), the SS band displays a hole-like dispersion centered at the $\bar{M}$ point, accompanied by a relatively flat intensity distribution near *E*_F_. High-resolution measurements near *E*_F_ (Fig. 2J) reveal that the SP band remains pinned to *E*_F_ over a wide *k* range of approximately ±0.2 Å^−1^ around the $\bar{M}$ point. Numerical fits of this region (Fig. 2K) further corroborate the flat dispersion (see also text S3). These results demonstrate that the SS band forms a 2D extended SP at the $\bar{M}$ point, enhancing the DOS at *E*_F_.

### Origin of the SP band dispersion

**Fig. 3.**
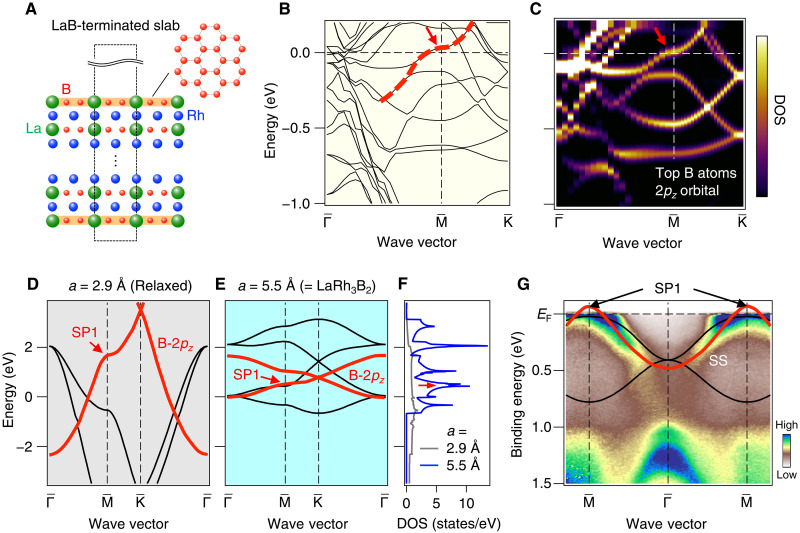
First-principles calculations reproducing the SS band. (**A**) LaB-terminated slab model used for band calculations. (**B**) Calculated band dispersion along the $\bar{\text{ΓMK}}$ cut, highlighting the SP feature (red dashed curve). (**C**) DOS projected onto the 2*p_z_* orbitals of the topmost boron layer, with arrows marking the SPs. (**D** and **E**) Calculated band structures for an isolated honeycomb boron layer with *a* = 2.9 Å (relaxed lattice constant) and *a* = 5.5 Å (nearly equal to that of LaRh_3_B_2_), respectively. Red curves indicate the boron-2*p_z_*–derived bands. (**F**) Calculated total DOS for the relaxed (gray) and expanded (blue) lattices in (D) and (E). A red arrow indicates the DOS peak of SP1. (**G**) Comparison of experimental ARPES data along the $\bar{\text{ΓM}}$ cut (gray scale) with the calculated band of the expanded honeycomb lattice, shifted by −0.45 eV to match the experimental result.

Having established that the SS band forms a SP, we next address its origin. To this end, we performed band-structure calculations using a slab model to focus on the surface state. We considered two possible surface terminations, LaB and Rh, and found that the LaB-terminated slab model (Fig. 3, A and B) reasonably captures the main experimental features (see also text S4). Although the calculated band structure along the $\bar{\text{ΓMK}}$ cut in Fig. 3B appears complex because of the finite size effect and multiple bands, it nonetheless reveals an SP near *E*_F_ whose dispersion (red dashed curve) matches that of the SS band. Analysis of the orbital contribution (Fig. 3C) shows that this SP band primarily originates from the 2*p_z_* orbital of the top-layer boron atoms (see also text S5). While the B 2*p_z_* orbital exhibits hybridization with underlying states as evidenced by the spread of its spectral weight, this coupling likely plays a key role in stabilizing the honeycomb boron framework, yet it robustly retains the SP band character near *E*_F_. These findings suggest that the SS band and associated SPs are fundamentally governed by the geometry of the surface honeycomb boron lattice supported by the bulk crystal.

To simplify the model and highlight the essential low-energy band features, we next considered a single honeycomb boron sheet. Figure 3D shows its band structure calculated with the relaxed in-plane lattice constant (*a* = 2.9 Å). The result resembles that of graphene; there is a σ band of *sp*^2^ hybrid orbital topped at $\bar{\Gamma }$ at *E*_B_ ∼ 2 eV and a π band of 2*p_z_* orbital forming a Dirac cone at the $\bar{K}$ point and an SP at the $\bar{M}$ point. However, compared to graphene, the lower band filling leads to a metallic band structure. Because the honeycomb boron lattice in LaRh_3_B_2_ is highly (nearly twice) expanded compared to the relaxed lattice, we also performed calculations with this expanded lattice constant *a* = 5.5 Å (Fig. 3E). While the overall structure is altered mainly because of the decomposition of the *sp*^2^ hybrid orbital to the 2*s* orbital (outside this energy window) and 2*p*-orbital bands (black curves), the 2*p_z_*-derived band remains present. Crucially, the 2*p_z_* bandwidth is dramatically reduced; the energy distance between the 2*p_z_* band bottom at the $\bar{\Gamma }$ point and the lower SP (labeled SP1) decreases from ∼4 eV in the relaxed lattice (Fig. 3D) to ∼0.6 eV in the expanded lattice (Fig. 3E), which is a factor-of-6 reduction. Consequently, the bandwidth becomes comparable to that of *d*-orbital materials such as cuprate superconductors and kagome metals, positioning it as a promising platform for studying strong correlation effects. This narrowing also compresses the DOS along the energy axis, yielding pronounced SP-induced peaks in the DOS (Fig. 3F, red arrow).

Figure 3G compares the experimental data with the calculated bands for *a* = 5.5 Å. Despite the simplicity of model, the 2*p_z_*-derived boron band shows a good agreement with the SS band when shifted by −0.45 eV, confirming its honeycomb boron lattice origin. The implied electron donation to the surface boron atoms may help stabilize the honeycomb boron structure (*15*) synergistically with the aforementioned interfacial coupling. Notably, the experimental SP band is flatter around the $\bar{M}$ point than the calculation. This observation suggests that the extended SP nature arises not only from the lattice expansion but also from additional mass renormalization mechanisms—likely many-body interactions (see text S6).

### Broken rotational symmetry observed by STM

**Fig. 4.**
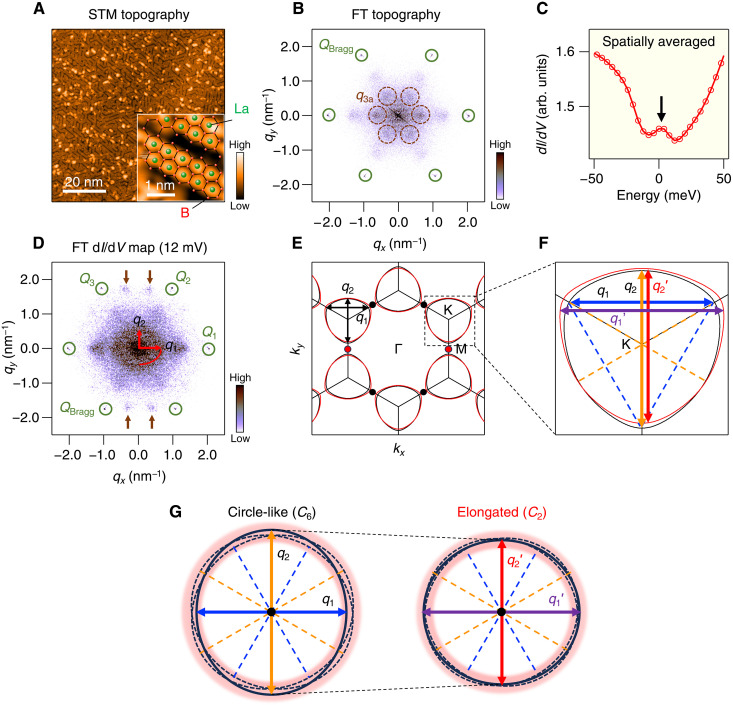
Broken rotational symmetry revealed by STM. (**A**) Topographic STM image of the cleaved LaRh_3_B_2_ surface. The inset shows an enlarged view highlighting the triangular arrangement of La atoms (green spheres). (**B**) FT topograph. Green circles mark the Bragg peaks, while brown circles denote the 3*a*_0_ short-range order. (**C**) Spatially averaged differential conductance (d*I*/d*V*) spectrum with a peak (arrow) slightly above *E*_F_, consistent with a VHS. (**D**) FT d*I*/d*V* map, showing the ellipse-like scattering pattern (red curve, *q*_1_, and *q*_2_). Brown arrows highlight peaks associated with the 3*a*_0_ order. STM setup condition (*V*/*I*): (A) 200 mV/1 nA; (inset) −100 mV/50 pA; (C and D) 200 mV/1 nA with a lock-in modulation of 5 mV. (**E** and **F**) Calculated energy contours and an enlarged view, respectively, from a tight-binding model just above the SP (+0.03 eV). Black curves represent the unmodulated band, while red curves include anisotropic hopping. Arrows and dashed lines represent origins of scattering vectors *q*_1_ and *q*_2_. (**G**) Schematic QPI patterns from the scattering channels for original (*q*_1_ and *q*_2_) and modulated (*q*_1_′ and *q*_2_′) Fermi pockets shown in (F).

To further examine the structural and electronic properties, we performed STM experiments, which complement the ARPES data by providing the atomic-scale real-space image of the cleaved surface and information on the unoccupied states. A representative topographic image of the cleaved surface (Fig. 4A) shows an atomically flat region with no observable steps over hundreds of nanometers (see also text S7). This area shows a mosaic parquet–like pattern comprising 1D chain-like features that extend only several nanometers (Fig. 4A, inset). Each chain consists of a triangular array of La atoms (green spheres in the inset), consistent with the LaB-terminated surface except that one-third of the La sites are absent, possibly causing the observed energy shift of the SS band. The Fourier-transformed (FT) topograph (Fig. 4B) exhibits a hexagon defined by sharp Bragg spots (*Q*_Bragg_) and additional diffuse spots at ~1/3 *Q*_Bragg_. These diffuse spots arise from the 3*a*_0_ periodic arrangement (*q*_3*a*_) of La vacancies. Because the chain-like domains are randomly oriented by ±120°, their superposition produces six *q*_3*a*_ spots.

A spatially averaged *dI*/*dV* spectrum in Fig. 4C acquired in the same field of view as Fig. 4A using a 600 × 600 grid shows a peak slightly above *E*_F_, consistent with a VHS (see texts S8 and S9). Whether this unusual electronic structure induces exotic phenomena is a central question. An essential clue is revealed in the FT spectroscopic map at an energy near the VHS peak (+12 mV), as shown in Fig. 4D. Unlike the nearly *C*_6_-symmetric topographic pattern (Fig. 4B), the intensity distribution in Fig. 4D exhibits clear *C*_2_ symmetry. Notably, rotational symmetry breaking is evident in the ring-like quasiparticle interference (QPI) pattern at the center, which becomes elongated and elliptical (see red curve). This rotational symmetry breaking is not an artifact of tip effects but is an intrinsic feature (see text S10). One might speculate that this anisotropic QPI pattern arises solely from the intrinsically 1D nature of the chain-like topography. However, such a structural origin alone cannot fully account for the present observations. This is because the scanned area of Fig. 4D (identical to that of Fig. 4B) contains 120°-rotated chain domains so that a purely structure-driven QPI pattern would be a superposition of three ellipses rotated by 120°, akin to the appearance of six *q*_3*a*_ spots in Fig. 4B. Since the QPI data in Fig. 4D displays a global uniaxial distortion despite this structural averaging, another mechanism is required to understand the observation. The most likely scenario is that the electronic system plays a dominant role in driving the symmetry breaking, indicating an electronically driven nematic state. The linearly combined component of *q*_3*a*_ with the Bragg peaks is strengthened along *Q*_1_ due to the emergence of the nematic electronic state (see arrows). These observations collectively support the presence of an electronically driven nematic instability.

To explore the origin of this *C*_2_-symmetric QPI pattern, we constructed a tight-binding model (see text S11 for details). First, we consider the case without nematicity. The black contours in Fig. 4E represent energy surfaces slightly above the SP, near the bias voltage of Fig. 4D. These surfaces contain rounded, triangular pockets centered at the K point, which are overall *C*_6_ symmetric. The ring-like QPI pattern arises primarily from the intrapocket scattering channel. As seen from an enlarged view of one pocket (Fig. 4F), its triangular shape leads to a small but finite difference in the length of the scattering vector between the horizontal (*q*_1_) and vertical (*q*_2_) directions. While this anisotropy creates a slightly distorted QPI pattern as shown by a solid black oval in Fig. 4G (left), the superposition of 120°-rotated ovals arising from the intrapocket scattering at different K points results in a circle-like pattern in total, keeping the *C*_6_ symmetry.

Next, to incorporate the nematicity, we introduced a *C*_2_-symmetric correction in hopping parameters that selectively lowers the energy of two of the six M-point SPs (Fig. 4E, red dots). As shown by the red curves in Fig. 4 (E and F), the resulting deformation appears only near those two M points, creating a unidirectional distortion of the triangular pockets along the vertical axis. Hence, in Fig. 4G (right), only the vertical components (*q*_2_) are greatly compressed, whereas the horizontal components (*q*_1_) remain nearly unchanged, yielding the experimentally observed uniaxial QPI.

To summarize, rotational symmetry breaking captured by STM is reasonably reproduced by our tight-binding model for honeycomb lattice based on the SS band having SP at *E*_F_ observed by ARPES. On the basis of this agreement, we conclude that the SS band plays a key role in realizing the rotational symmetry breaking. Furthermore, since any signature of rotational symmetry breaking, such as anisotropy in electrical resistance or crystal lattice, has not been reported for bulk both in normal and superconducting states (*20*–*22*), it is natural to consider that the observed rotational symmetry breaking is of surface origin.

## DISCUSSION

We now turn to the origin and implications of the observed electronic nematicity. Various Fermi-surface instabilities at van Hove filling have been predicted in honeycomb and kagome lattices (*11*, *12*, *14*, *23*–*26*), and one well-established way to relieve such instabilities is the opening of an energy gap, for example, through a charge-density wave. On the other hand, by integrating the ARPES band structure with the STM-based QPI findings, we propose that a momentum-selective (or momentum-dependent) shift of SPs primarily drives the nematic order and effectively suppresses the DOS at *E*_F_ (see text S11). This shift leads to the band-structure deformation characterized by *q* = 0 (Fig. 4, E and F), which resembles that induced by Pomeranchuk instability (*27*). Unlike the more familiar SP-driven charge density–wave instability at finite *q*, where the translational symmetry is broken, the *q* = 0 character of electronic nematicity represents a fundamentally different mechanism. This unusual state partly stems from the highly expanded boron lattice, which substantially narrows the bandwidth and thereby enhances the correlation effect. In addition, the absence of parallel Fermi-surface segments arising from the extended SP formation and the distortion of the hexagonal Fermi surface (Fig. 2B) can suppress the nesting-driven 2 × 2 density-wave order, thereby favoring nematicity as a dominant pathway (*28*).

Electronic nematicity has emerged as a central issue in modern condensed matter physics, especially in correlated electron systems such as high-temperature superconductors and kagome metals. Compared to earlier reports of nematicity, LaRh_3_B_2_ offers several unique features. First, its honeycomb boron lattice hosts SPs at three nonequivalent M points, in contrast to the square lattices of cuprates (*29*–*32*) and iron-based superconductors (*33*, *34*) where SPs typically appear at two different momenta, for instance, (π, 0) and (0, π) points in cuprates. The presence of more SPs in a honeycomb network can yield a richer variety of symmetry-breaking states (*12*). Second, unlike other hexagonal systems such as kagome metals, *A*V_3_Sb_5_ (*A* = K, Rb, and Cs), *A*Ti_3_Bi_5_ (*A* = Rb and Cs), and ScV_6_Sn_6_ (*35*–*38*), which have multiorbital band structures, the boron honeycomb band in LaRh_3_B_2_ is comparatively simpler. This simplicity should facilitate theoretical studies of the microscopic origin of nematicity. Third, whereas prior research on nematicity focused mainly on strongly correlated *d* electron systems, the honeycomb boron lattice involves *p* electrons, which are typically considered weakly correlated. Nematicity associated with the Pomeranchuk instability has been theoretically predicted for a *p* electron system, specifically for doped graphene at van Hove filling (*12*, *13*), but has not been experimentally realized, likely because of its weak correlations. In our work, the synergy between lattice expansion and many-body interaction effectively enhances the electron correlations giving rise to nematicity despite the *p*-electron character of the system. Identifying this distinct platform, which combines these unique properties, is therefore a critical step forward.

Last, our findings suggest a promising route to stabilizing unconventional 2D electronic states. Through a complementary combination of ARPES, STM, and density functional theory calculations, we identified a distinctive 2D electronic state originating from the highly expanded honeycomb boron lattice at the surface of LaRh_3_B_2_. This structure would be difficult to stabilize as a free-standing monolayer as it differs substantially from previously fabricated borophene sheets on metals. Our results thus imply that exposing a peculiar 2D lattice embedded in a 3D crystal is an effective way to realize otherwise unstable 2D electron systems. Even within the broader LaRh_3_B_2_ family (*RT*_3_*X*_2_), the wide range of elemental substitutions (*R*, *T*, and *X*) offers a versatile platform pursuing exotic phenomena by tuning the electronic structure of such 2D electron systems. We also captured a sign of chirality (see text S7), implying the potential of coexisting electronic orders and their interplay. Furthermore, this system can be regarded as a combination of 2D layers and 3D bulk, where the 2D electronic states are not fully isolated. This unique coupling would offer a promising platform for exploring exotic phenomena driven by interfacial effects.

## MATERIALS AND METHODS

### Sample preparation

Single-crystalline samples were grown by the Czochralski pulling method in a tetra-arc furnace, following the procedure described in a previous study (*39*). The occurrence of bulk superconductivity in our crystals (*20*) rules out the presence of a large amount of La vacancies throughout the bulk because such imbalance in stoichiometry is known to suppress bulk superconductivity in LaRh_3_B_2_ (*16*, *21*, *40*). Therefore, the La vacancies are most likely induced on the surface in the cleavage process. A main influence of these vacancies would be a modification of the local carrier concentrations at the surface. Our Bader charge analysis, which attributes electron density of crystals to each atom (*41*), indicates that La atoms act as electron donors. As a result, La vacancies cause an electron deficiency and an upward energy shift of the surface bands relative to the calculation for a stoichiometric slab. This effect has an important role in fine tuning the VHS to the vicinity of *E*_F_. While the La vacancies generate a 3*a*_0_ periodicity, this is short range with a typical length of only several nanometers. Hence, it does not produce visible folding effects in the global band structure observed by ARPES.

### ARPES experiments

ARPES measurements were carried out at BL5U (UVSOR) using an MBS A1 analyzer and at BL-2A and BL-28A (Photon Factory, KEK) using Scienta-Omicron SES2002 and DA30 analyzers, respectively. Unless otherwise specified, measurements used linearly polarized photons of *h*ν = 60 eV, and the energy resolution was set between 20 and 30 meV. Samples were cleaved in situ in an ultrahigh vacuum (<1 × 10^−10^ Torr), and *E*_F_ was referenced to that of a gold film electrically contacted to the sample holder. All ARPES data were taken at *T* = 7 K.

### Band calculations

First-principles band-structure calculations were carried out by using the QUANTUM ESPRESSO code package (*42*, *43*) with generalized gradient approximation and Perdew-Burke-Ernzerhof exchange-correlation functional (*44*). The projected-augmented-wave (PAW) method (*45*) was used for the pseudopotentials. Spin-orbit coupling was not included but scalar relativistic effects were accounted for via scalar-relativistic potentials. For bulk band calculations, we used a plane-wave cutoff energy of 100 Ry and an 8 × 8 × 12 *k*-point mesh. The crystal structure of LaRh_3_B_2_ was optimized using variable-cell relaxation, with convergence thresholds of 10^−5^ Ry for the total energy and 10^−4^ Ry/Bohr for the forces. Phonon calculations were performed using density functional perturbation theory implemented in the QUANTUM ESPRESSO (*42*, *43*). The threshold self-consistency is set to 10^−15^ with a *q*-grid of 4 × 4 × 2. For slab calculations, we constructed a supercell comprising six bulk unit cells in thickness, separated by a vacuum layer of over 30 Å to eliminate interslab interactions. A plane-wave cutoff energy of 50 Ry and a 4 × 4 × 1 *k*-point mesh were used for these slab models.

### STM experiments

Samples were cleaved under ultrahigh vacuum at ~15 K and immediately transferred into the cryogenic STM stage. All the STM data were acquired at 4 K under zero external magnetic field. Tungsten tips were calibrated on an Au(111) surface before use. The differential conductance (d*I*/d*V*) was measured using a standard lock-in technique with a bias modulation frequency of 961 Hz. The lattice constant estimated from the Bragg spots in the FT topographic image (Fig. 4B) is ~5.6 Å, which is almost the same as the bulk value (*16*, *21*).

## Supplementary Materials

### This PDF file includes:

Supplementary Text S1 to S11 Figs. S1 to S11 References
