# Supplementary material for: Realization of strongly correlated 2D honeycomb boron
Source: Sci Adv. 2026 Jul 1;12(27):eaee3116. doi: 10.1126/sciadv.aee3116 (PMC13455447; doi:10.1126/sciadv.aee3116)
Supplement: Supplementary file 1 [file sciadv.aee3116_sm.pdf]

Science Advances
AAAS

Supplementary Materials for

Realization of strongly correlated 2D honeycomb boron

Takemi Kato *et al.*

Corresponding author: Takemi Kato, t.kato@oist.jp; Kosuke Nakayama, k.nakayama@arpes.phys.tohoku.ac.jp; Yoshinori Okada, yoshinori.okada@oist.jp; Takafumi Sato, t-sato@arpes.phys.tohoku.ac.jp

Sci. Adv. **12**, eaee3116 (2026)
DOI: 10.1126/sciadv.aee3116

This PDF file includes:

Supplementary Text S1 to S11
Figs. S1 to S11
References

Supplementary Text S1

Difference between the present and previous work

The SS band had not been observed by a previous work by Iida *et al* (*19*). This difference originates from the time-dependent change in the spectral feature due to radiation damage during measurements. Figs. S1A,B compares Fermi surface maps taken under the same experimental setup but on freshly cleaved surface (Fig. S1A) and after several hours of measurement (Fig. S1B). Figure S1B reveals that the hexagonal Fermi surface formed by the SS band seen in Fig. S1A completely disappears. In addition, double triangular electron pockets centered at the K points (dashed triangles; see Fig. S1C for their electron-like characters) and a circular intensity at the $\bar{\Gamma}$ point from the hole band become prominent (see also Fig. S2M). These features are in good agreement with the previous results shown in Fig. S1D (*19*). This demonstrates that the electronic structure changes from a fresh state with clear SS bands (our main finding) to a state that aligns better with the bulk band calculations, and ref. (*19*) only observed the latter state where the SS band had already vanished. This is likely caused by the different measurement conditions: the He discharge lamp used in ref. (*19*) introduces residual gases making vacuum worse and has a lower photon flux (compared to the synchrotron light in this work) requiring longer measurement time, both of which accelerate the degradation of surface and disappearance of the SS band.

Supplementary Text S2

Photon-energy dependence of the surface states and bulk bands

We have performed normal emission measurement using soft x-ray to estimate the inner potential V_0. Fig. S2B shows E_B-k_z map exhibiting a periodic dispersion along with that of the Brillouin zone shown in Fig. S2A. Comparison with the calculation in Fig. S2C shows a reasonable agreement although there is finite energy difference to some extent. From these results, we estimated $V_0 = 26$ eV, leading to the relationship between $h\nu$'s and k_z's as shown in Fig. S2D. With this V_0 value $h\nu = 60$ eV probes the middle of ΓA ($k_z \sim 0.6\pi$). It is noted that, however, the SS band remains unchanged across the entire photon energy range of $h\nu = 30$–90 eV covering ΓA as shown in Fig. S2E–K. While the intensity distribution of several bands varies with photon energy, the dispersion of SS band remains unchanged, confirming its 2D character.

Bulk band calculations in Fig. S2L show that, while Rh kagome lattice generates VHSs as denoted by "VHS", these VHSs are located far (more than 1.5 eV) above E_F (*21*). Therefore, they are unlikely to contribute to low-energy electronic properties and strongly correlated behavior. Also, the contribution of other bulk electronic structure is considered negligible. This is because, while another bulk-derived VHS from the honeycomb boron lattice exists at the L point ($k_z = \pi$) at the top of the hole-like band highlighted by blue dashed line in Fig. S2I (see also Fig. 2C), it is located ~0.2 eV below E_F. Moreover, other bulk bands with La-derived orbital characters cross E_F, as observed by ARPES, e.g., near the A point (see an electron-like band indicated by red arrow in Fig. S2I), but they exhibit conventional dispersion without singularities. These results support our conclusion that correlation effects identified near E_F are intrinsic to the 2D surface state and not driven by bulk-derived states.

While not all the bulk bands were observed in Fig. 2C possibly due to matrix-element effect of photoelectron intensity, we could indeed observe bulk bands near E_F by varying experimental conditions. For instance, we observed both electron- and hole-like bands near the $\overline{\Gamma}$ point by changing $h\nu$ (k_z), as shown in Fig. S2I,M, respectively. In addition, by azimuthally rotating the sample by 90˚ and measuring to higher binding energies, we found good agreement between ARPES results (Fig. S2M) and bulk band calculation (Fig. S2N), such as hole-like dispersion topped near E_F at the $\overline{\Gamma}$ point (indicated by red arrow), a flat band at E_B ~ 1.0 eV (magenta arrow), and electron-like feature at E_B ~ 1.7 eV around the $\overline{\Gamma}$ point (black arrow). These observations suggest that our calculations reasonably capture several bulk bands. It is noted that the hole-like dispersion topped at E_B ~ 0.5 eV at the $\overline{\Gamma}$ point (blue arrow), which is not reproduced by the bulk band, can be seen in the slab calculation in Fig. S2O and it could be surface state.

It is also noted that, away from E_F, bulk electronic states can interact with the SS band. Notably, as shown in Fig. S2F, the intensity of SS band is suppressed near the $\overline{\Gamma}$ point where it overlaps with a hole-like bulk band. While this suggests hybridization, as seen in Bi(111) surface (*46*), the hybridization area in momentum space is primarily located away from E_F and does not affect the renormalization effect found for saddle-point VHS at the $\overline{M}$ point. It is worth noting that the observation of a hole-like band at the zone center in Fig. S2F ($h\nu$ = 40 eV that probes k_z ~ 0), in contrast to the electron-like character in Fig. S2I ($h\nu$ = 70 eV that probes k_z ~ π), is associated with the difference in $h\nu$ and k_z, supporting the bulk nature of the band observed at the zone center.

Supplementary Text S3

<u>SS band dispersion near E_F</u>

The data in Fig. 2K were obtained by fitting each EDC with a single Lorentzian-type peak, incorporating the FD function and the instrumental resolution in the fitting procedure. As seen in the main text, the SS band exhibits an extended SP dispersion very close to E_F. This near-E_F positioning could be influenced by many-body interactions, including self-induced renormalization effect caused by its own high DOS near E_F (*11*, *47*, *48*).

To examine the detailed band dispersion of the SS around E_F, we divided ARPES spectra around the $\overline{M}$ point obtained at T = 30 K by the Fermi-Dirac (FD) distribution function convoluted with a Gaussian resolution function (Fig. S3A). We then extracted the band dispersion (red circles) by numerical fitting. The result shows that the hole-like dispersion is nearly topped at E_F (~10 meV above E_F), consistent with the peak near E_F in the energy-distribution curve (EDC) at the $\overline{M}$ point (Fig. S3B).

Supplementary Text S4

<u>Termination dependence of calculated band dispersion</u>

To clarify the origin of the SS band, we performed slab-model calculations with two possible surface terminations; LaB and Rh, based on the bulk crystal structure of $LaRh_3B_2$. Figures S4A,B show schematic views of these two slab models. Figures S4C,D display the corresponding band structures. In the LaB-terminated model (Fig. S4C), a SP dispersion appears in the vicinity of E_F

at the $\overline{\mathrm{M}}$ point. By contrast, no such dispersion is found near E_F for the Rh-terminated slab (Fig. S4D). Comparison with the ARPES data indicates that the LaB-terminated slab model accounts more plausibly for the observed SS band. Finally, for the isolated honeycomb boron sheet, we minimized the total energy to determine the in-plane lattice constant, thereby obtaining the fully relaxed configuration.

Supplementary Text S5

DOS contribution of La atoms in the surface layer

We also examined the contribution of La lattice in the surface layer. Figure S5 shows density of states (DOS) projected on (A) top La and (B) top B atoms calculated for the slab model shown in Fig. 3A. Although the surface La atoms have a finite DOS at the saddle point (indicated by red arrow in Fig. S5A), their contribution is much suppressed compared to that of the surface B atoms (Fig. S5B). This confirms that the B-2*p* orbitals of the surface honeycomb lattice are the key source of the VHS.

Regarding the surface reconstruction, even if the topmost La atoms are reconstructed, the electronic structure derived from the boron lattice can still be robustly captured by ARPES and STM. Similar situations were reported, for example, in an STM study on a kagome superconductor RbV_3Sb_5 (*49*). In the case of RbV_3Sb_5, although the surface Rb atoms exhibit four types of reconstructions, QPI from an electron band of buried Sb atoms has been robustly observed on all the reconstructed surfaces. Similarly, in $LaRh_3B_2$, while the surface reconstruction of La atoms can induce energy shift (see also the Method section), the main feature of boron lattice (i.e., the SS band) has been clearly captured by ARPES and STM.

Supplementary Text S6

Dispersion kink and possible many-body interactions

The discontinuities in the SS band intensity in Figs. 2D,F (along **k** cuts parallel to the $\overline{\Gamma\mathrm{K}}$ line) originate from hybridization with the electron bands centered at the $\overline{\mathrm{K}}$ point shown in Figs. S3B,C. However, the dispersion kink along the $\overline{\Gamma\mathrm{M}}$ cut, where SPs exist, must have a different origin because these electron bands do not appear along this cut. Namely, we have selected the $\overline{\Gamma\mathrm{M}}$ cut for the analysis of the dispersion kink to avoid hybridization with other bands. Hybridization between the bands shown in black and red curves (Fig. 3G) is also unlikely to account for the existence of the kink, because of their different orbital characters (note that p_x/p_y for black and p_z for red cannot hybridize with each other). The robust appearance of the kink by varying $h\nu$ (Fig. S6A) also rules out a band overlap scenario because the intensity ratio is generally $h\nu$ dependent when there are two different bands. From these considerations, electron-phonon coupling is the most plausible origin of the kink.

To further support electron-phonon coupling origin of the kink, we have performed more quantitative analyses. Figures S6B–D show the band dispersion extracted by numerical fitting, the MDC full width at half maximum (FWHM), and the real part of the self energy (ReΣ), respectively. Marked decrease in FWHM (Fig. S6C) and a broad peak at $E_B \sim 0.05$ eV in ReΣ (Fig. S6D)

represents an energy scale of electron-phonon coupling. We estimated the coupling constant λ to be $\lambda \sim 0.7$ from the first derivative of ReΣ at around E_F. It is noted that, while a large curvature in the SS band dispersion near E_F makes it difficult to estimate a correct bare band shape, our results should still represent an energy scale linked to the electron-mode coupling, and it is worth discussing its origin by comparing with phonon calculation. In this context, since our phonon calculations for bulk (Figs. S6E,F) reveal dominant boron-derived phonon modes around an energy scale of ~0.05 eV (see Fig. S6G for corresponding modes at the M point), a coupling with boron modes is the leading candidate for the origin of the kink, while definitive mode identification would require future ARPES studies with higher precision. Overall, these results indicate that the large DOS near the extended SP may be further enhanced by many-body effects, as schematically illustrated in Fig. S6H.

Supplementary Text S7

Another termination observed by STM

In our STM measurements, we observed a termination that can be attributed to the Rh layer. The representative results are shown in Fig. S7A, where a clear step-and-terrace feature is visible. The terraces seen at left and right bottom of Fig. S7A correspond to the ordered LaB-terminated surface discussed in the main text. The other terrace dominant in Fig. S7A has a height that is ~0.8 nm lower than the LaB surface (see the inset for a height profile across a step). This step height is approximately 2.5 times the lattice constant *c*, consistent with the expected Rh-terminated surface. However, as seen from the enlarged topography in Fig. S7B, this Rh-terminated surface is disordered and mostly covered by nanoparticles, which has been similarly observed in a Rh-dosed surface on TiO_2 (*50*). This surface does not show even 1×1 Bragg peak in an FFT image, preventing us from further examining the electronic structure in this surface. This disorder on the Rh termination indicates that the clear ARPES intensity with VHS shown in the main text dominantly comes from the ordered LaB-terminated surface, and excludes dominant contributions from the disordered Rh-terminated surface to the observed rotational symmetry breaking.

Supplementary Text S8

Reproducibility of the observation of VHS

To confirm reproducibility of the dI/d*V* spectrum in Fig. 4C, we performed the same measurement in an independent field of view shown in Fig. S8A using a 280×280 grid. The spatially averaged d*I*/d*V* spectrum in this new region (Fig. S8B) consistently exhibits a peak in the vicinity of E_F, supporting our observation of VHS.

Supplementary Text S9

The other VHS in a d*I*/d*V* spectrum

According to our band calculation (e.g., Fig. 3E), the other saddle-point VHS (upper VHS shown in Fig. 1F–J) is located ~0.5 eV above SP1. In Fig. S9, we show a *dI*/*dV* spectrum for a wide energy range, where a peak structure can be found at ~0.5 eV as indicated by the arrow (it is noted that

we have not taken a spectrum mapping like Fig. S8 but a point spectrum at a clean location to avoid a possible damage to the STM tip at high bias voltages). This high-energy peak is consistent with the other VHS, further supporting the SS band formation derived from the boron honeycomb lattice.

Supplementary Text S10

Excluding extrinsic effects for observed rotational symmetry breaking

The nematicity observed via STM is intrinsic in nature rather than an artifact of an extrinsic tip effect or other instrumental factors. First, we address the possibility of scanner piezo distortion of the FFT image. This is quantitatively confirmed by comparing normalized line profiles along the Q_1, Q_2, and Q_3 direction (Fig. S10A): the intensity of the elliptical QPI is anisotropic and, importantly, the peak center is at a larger $|q|$ along the ellipse's major axis (Q_1) than along the other directions (see $|q|/|Q_n| \sim 0.3$; red shaded region), while aligning the Bragg peak center (blue shaded region). This indicates that the anisotropic QPI shape is not caused by extrinsic distortion during the scanning process. Second, tunneling matrix-element effects are naturally excluded. Although anisotropic electronic orbital shape at the top of the tip can weight tunneling along certain directions, they cannot change the absolute magnitude of $|q|$ determined by peak structure in FT images. The elliptical QPI feature (Figs. 4D,S10A) is therefore not attributable to the extrinsic tip effects.

Next, regarding the possible strain, we have assessed whether extrinsic strain could account for the observed QPI anisotropy. Since it is intrinsically difficult to determine the absolute uniform strain solely from STM measurements, we have evaluated maximum possible extrinsic strain by analyzing the mean Bragg wave vectors obtained by Fourier transformation of the topographic images. As a result, the possible strain range was estimated to be between −1.15 % and +1.17 % (note that this includes potential instrumental artifacts such as piezo creep, thermal drift, and scanner nonlinearity, setting the upper limit). To estimate the impact of this possible strain on the anisotropic electron hopping, we used an exponential decay model $e^{-3.37\times\delta}$ (δ is a relative change in bond length, which is established for π band of p_z orbital in graphene in Ref. (*51*)). Given $\delta \leq 0.01$, the change in the hopping parameter was estimated to be only ~3% at most. Since our tight-binding simulations require an 8% modification for the hopping term to reproduce the observed elliptical QPI ($|k_2|/|k_1| \sim 0.9$), we conclude that the extrinsic strain alone is too small to account for our experimental results.

Beyond the C_2-symmetric nematic behavior shown in Fig. 4, we also found a signature of chirality, which is analogous to the previously reported chiral electronic order in other systems but using STM (*52*). Figure S10B plots the energy dependence of the normalized Bragg-peak intensity of $I(Q_2)/I(Q_1)$ (blue) and $I(Q_3)/I(Q_1)$ (green). In a conventional case without exotic ordering, the ratios between any Bragg-peak intensities extracted from dI/dV maps would remain constant independent of energy. Our data, however, reveal a deviation from such a conventional behavior: the two ratios are not constant and show clearly different energy dependencies. This indicates that Q_2 and Q_3 become non-equivalent relative to Q_1, suggesting a broken symmetry beyond nematicity

where Q_2 and Q_3 are equivalent. Such non-equivalence may point to an underlying chirality, which is nontrivial to realize in a nominally 2D system. These observations imply that chirality may be associated with the substrate contribution. We propose that the spontaneously formed electronic hetero-interface between honeycomb boron lattice and the surface kagome metal offers a promising platform for exploring rich broken electronic symmetries, including both nematicity and chirality.

Supplementary Text S11

Details of the tight-binding simulation

To link our ARPES and STM results, we developed a tight-binding model for a honeycomb lattice including terms up to third-nearest neighbors (*53*). The parameters used are (e, t_1, t_2, t_3) = (0.33, −0.21, −0.09, 0.1) in the unit of eV. In Figs. S11A,B, we overlay the calculated band structure (black curves) on the experimental Fermi-surface map and high-symmetry cuts. The model captures both the warped Fermi surface and the extended saddle point near E_F. We note that Fig. S11B also exhibits an additional band flattening near E_F, consistent with the "kink" feature discussed in Fig. S6. Next, to simulate the observed anisotropy, we reduced t_1 by 8% for one of the three directions while the other two remained unchanged. As illustrated in Fig. S11C, this results in a distorted hexagon-like Fermi surface consistent with C_2-symmetric QPI and the ARPES intensity. Importantly, this deformation of Fermi surface is associated with a slight energy difference of VHSs at $\overline{\mathrm{M}}$ and $\overline{\mathrm{M}}$' points; namely, one VHS shifts upward while the other shifts downward, as shown in Fig. S11D. Because of this energy splitting, the VHSs are pushed away from E_F (see inset in Fig. S11D), which effectively removes the divergent DOS at E_F similar to the gap-opening case. Furthermore, the dI/dV spectrum with higher energy resolution and finer steps (Fig. S8B) reveals a dip-like feature near zero bias, experimentally supporting the suppression of the DOS at E_F.

While the introduction of anisotropic hopping results in only a small distortion in energy contour slightly above E_F and SP (+0.03 eV) (see red curve in Fig. S11E), its autocorrelation (Fig. S11F,G), which can be considered as an indicator of quasiparticle scattering, shows a clear rotational symmetry breaking represented by an ellipse elongated along k_x direction traced by a half white circle in Fig. S11G (note that this pattern is derived from intra electron scattering within the pockets at the $\overline{\mathrm{K}}$ point), in contrast to an isotropic circular intensity in the absence of anisotropic hopping (see Fig. S11F). The ratio between longer and shorter vectors of the ellipse along the $\overline{\Gamma\mathrm{M}}$ direction indicated by the red arrows (k_1 and k_2) is ~1 : 0.9, which is in good agreement with the STM result.

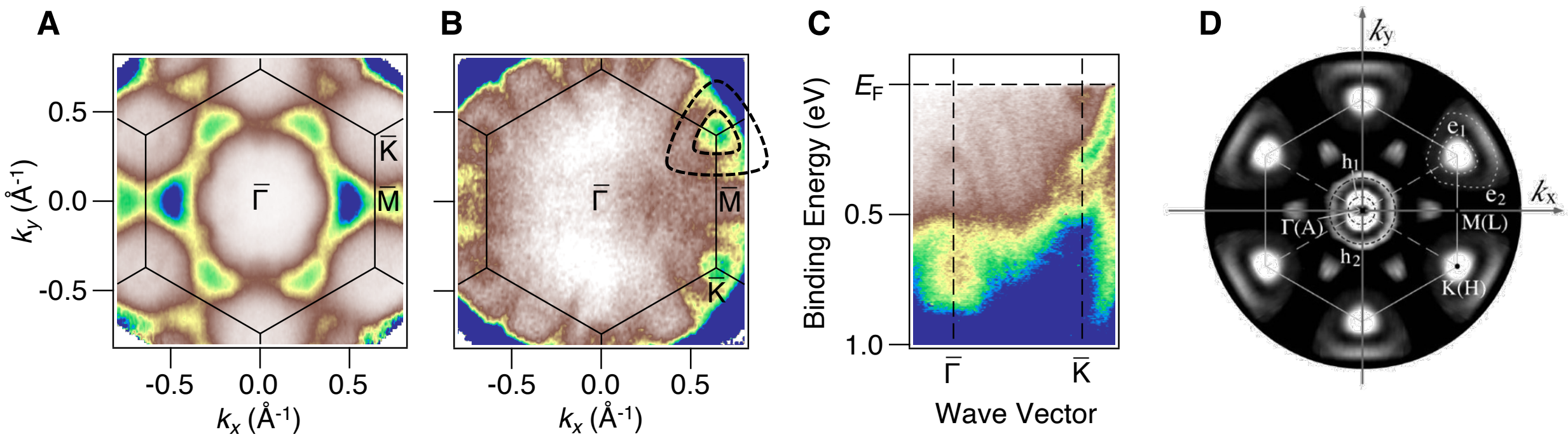

Fig. S1. Time-dependent ARPES intensity.

(A,B) Fermi surface mapping measured with $h\nu$ = 60 eV on freshly cleaved surface and aged surface, respectively. (C) Valence-band ARPES intensity along the $\overline{\Gamma K}$ cut for (B). (D) Fermi surface mapping in the previous work (*19*) measured with a He discharge lamp ($h\nu$ = 21.218 eV).

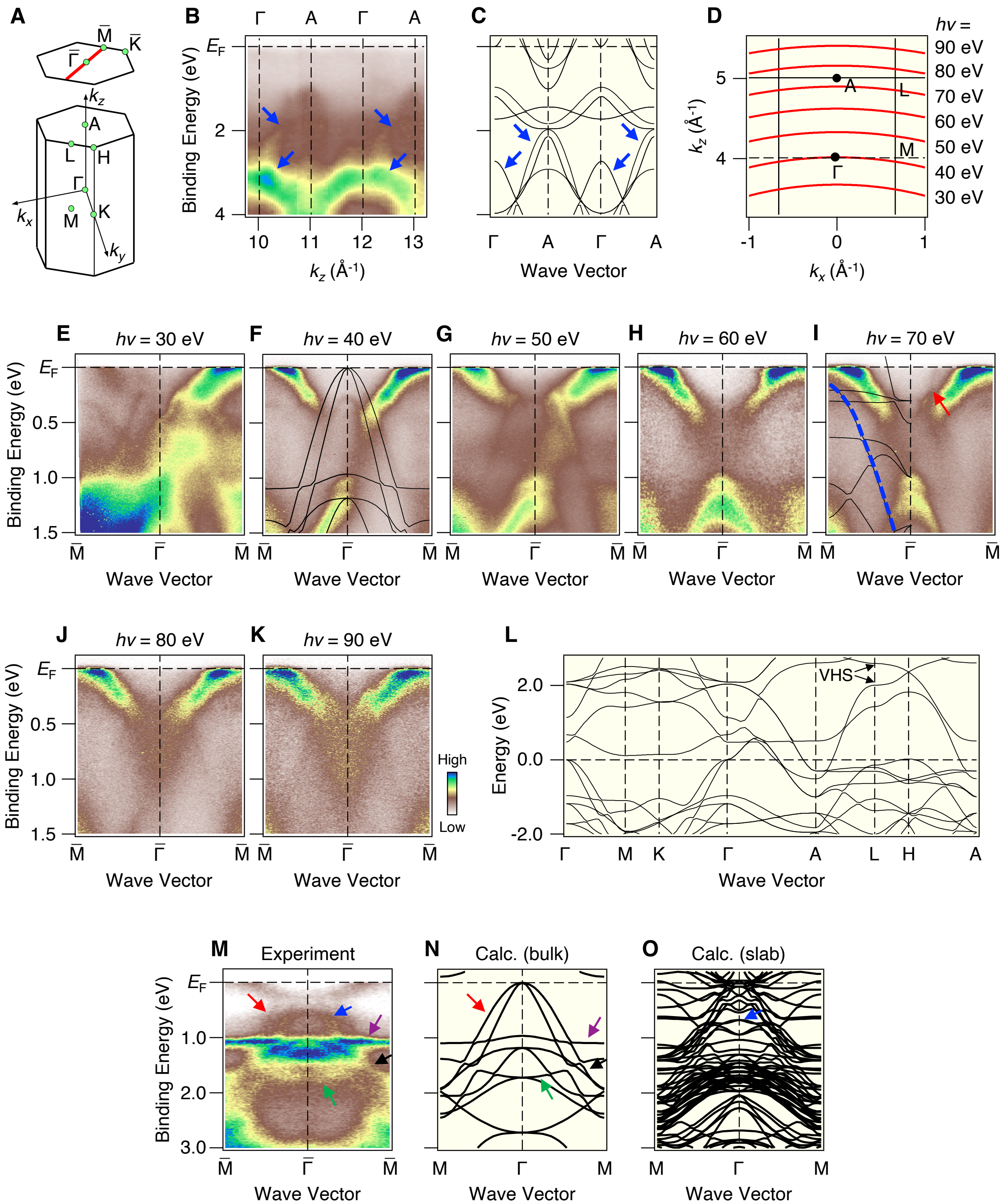

Fig. S2. Photon-energy dependence of band structure.

(A) Bulk and surface Brillouin zone of $LaRh_3B_2$. (B,C) Experimental E_B-k_z band dispersion obtained by normal emission ($h\nu$ = 350–650 eV) and corresponding calculation. (D) Probed k values for $h\nu$ = 30–90 eV in the Brillouin zone. (E–K) ARPES-intensity maps along the $\overline{\Gamma M}$ cut [red line in (A)] measured with $h\nu$ = 30–90 eV. Solid curves in (F) and (I) are calculated band

dispersion of bulk $LaRh_3B_2$ at k_z = 0 and π. (L) Calculated band dispersion along the high-symmetry lines for bulk $LaRh_3B_2$. (M) ARPES intensity along the $\overline{\Gamma M}$ cut measured with $h\nu$ = 60 eV for a sample that is rotated by 90° in-plane relative to that in (H). (N,O) Calculated band dispersion along the ΓM cut for bulk and slab models shown in Fig. 3A, respectively.

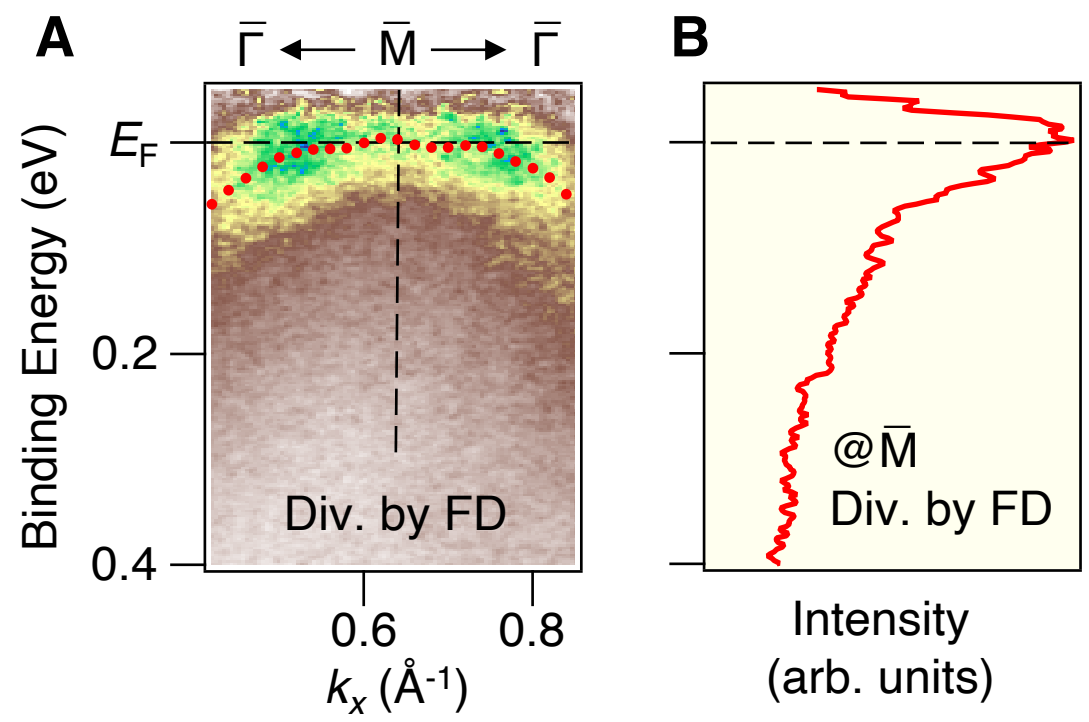

Fig. S3. Near-E_F ARPES spectra divided by the FD function.

(A) ARPES intensity around the $\overline{\mathrm{M}}$ point divided by the FD function. Red circles mark the extracted dispersion of the SS band, obtained via Lorentzian peak fitting. (B) EDC at the $\overline{\mathrm{M}}$ point, divided by the FD function, showing that the SS band is topped around 10 meV above E_F.

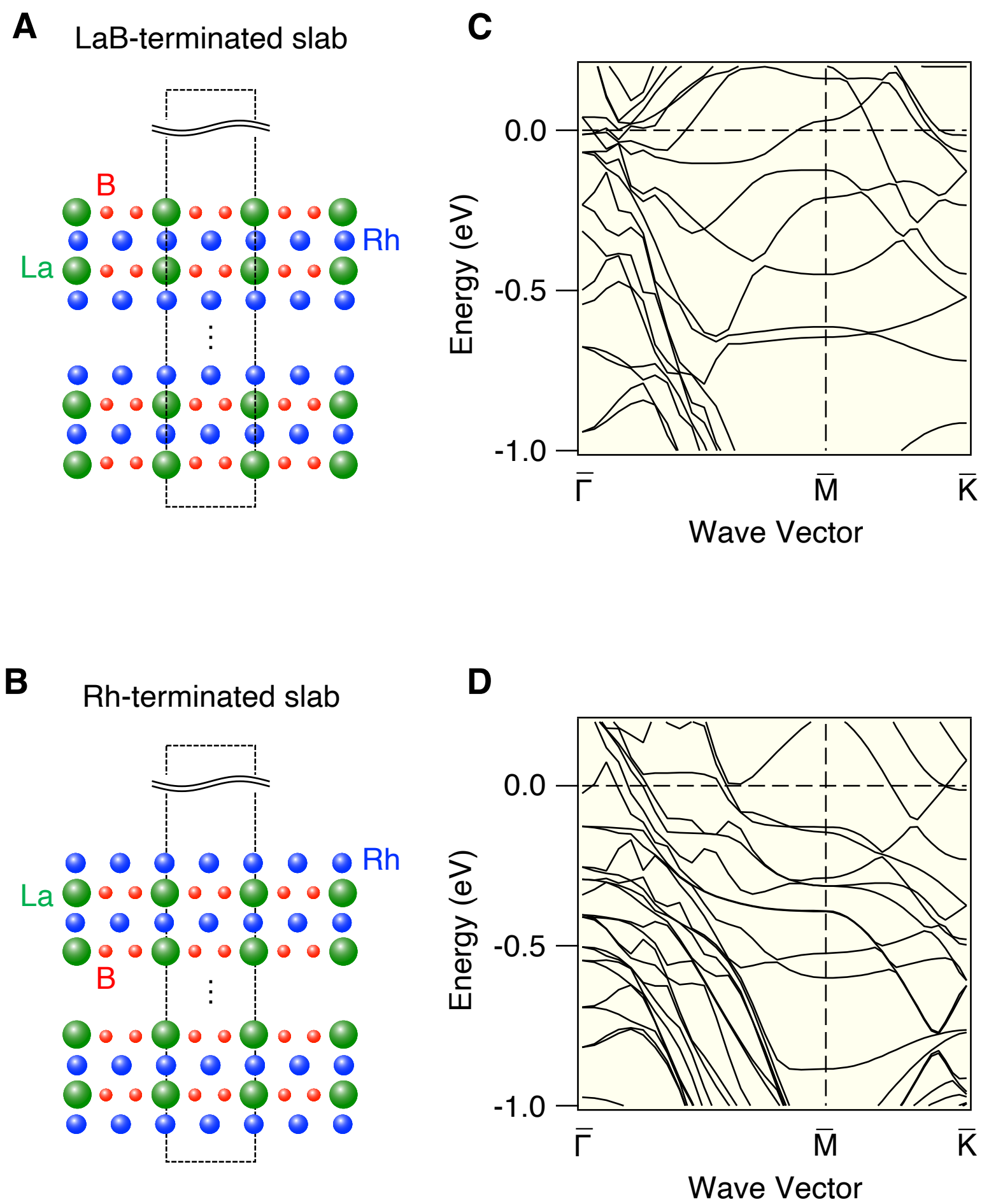

Fig. S4. Two slab models and calculated band dispersion.

(A,B) LaB- and Rh-terminated slab model, respectively, used for the theoretical simulation. (C,D) Calculated band dispersion for models (A) and (B), respectively. A saddle point emerges near E_F only in the LaB-terminated model (C).

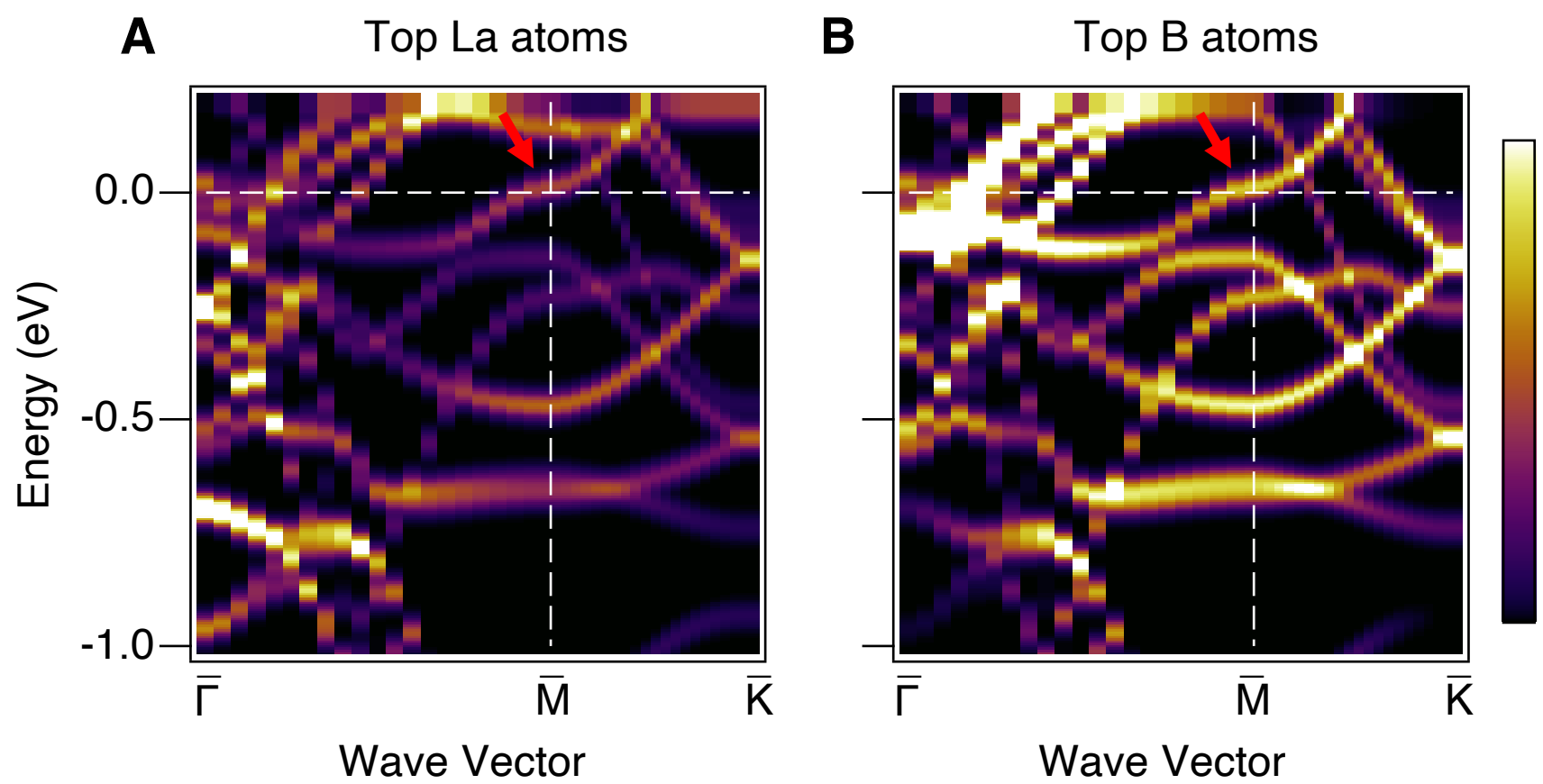

Fig. S5. Calculated DOS for the slab model projected on the topmost atoms.

(A,B) Calculated DOS for the slab model shown in Fig. 3A projected on the topmost La and B atoms, respectively, with arrows marking the saddle points.

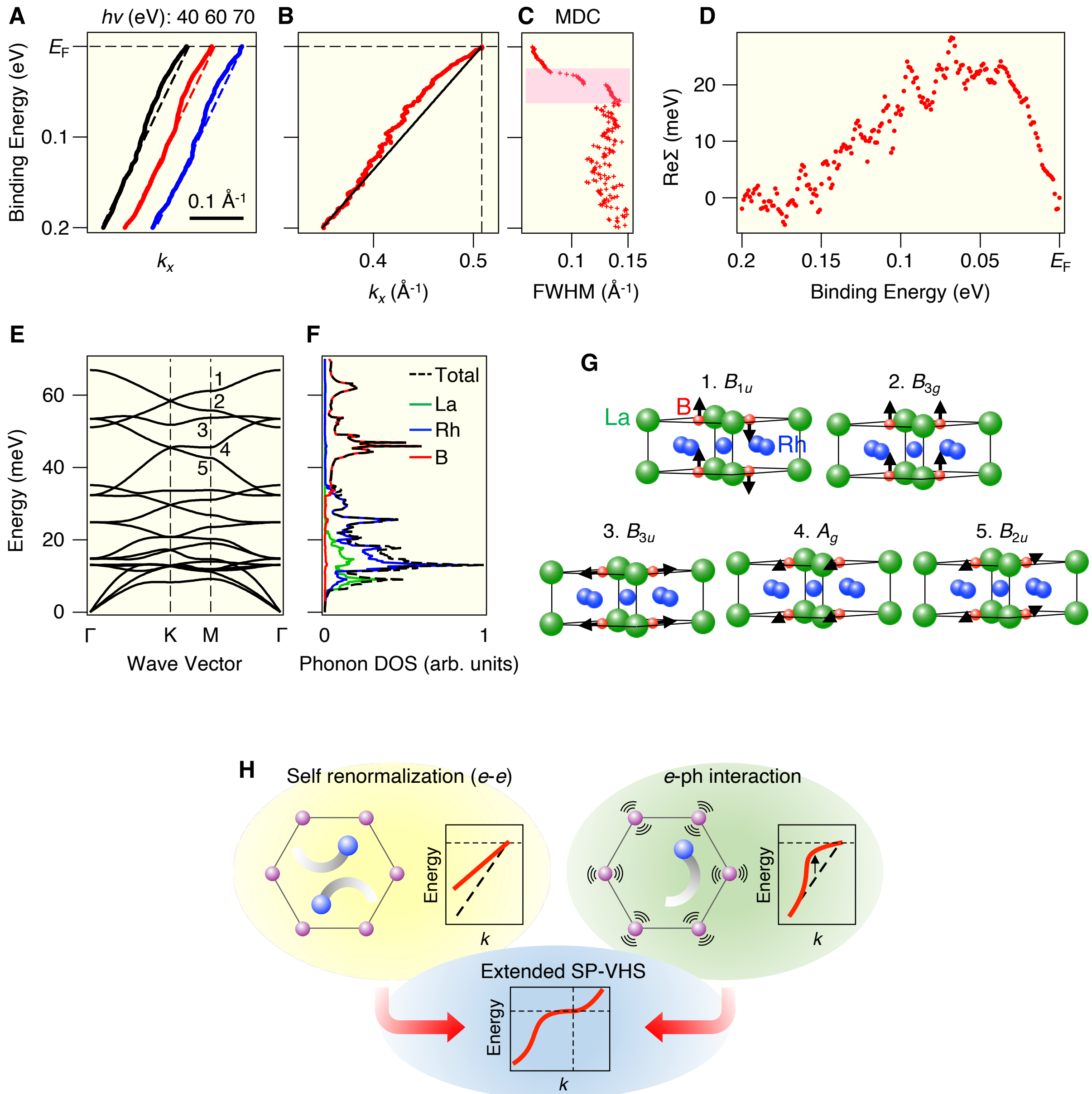

Fig. S6. Kink analysis and possible many-body interactions.

(A) Extracted dispersion of the SS band along the $\overline{\Gamma M}$ cut estimated by numerical fitting to MDCs for $h\nu = 40$ eV ($k_z \sim 0$; black), 60 eV (red), and 70 eV ($k_z \sim \pi$; blue). Dashed lines are shown to emphasize the kink and have same slope. (B) Same as (A) but measured with $h\nu = 60$ eV and higher energy resolution (ΔE = 20 meV). A black solid line is a bare band. (C) FWHM of the MDCs, showing a sharp drop at ~0.05 eV. (D) ReΣ obtained from (B). (E–G) Calculated bulk phonon dispersion and DOS for $LaRh_3B_2$ and corresponding phonon modes (1–5) at the M point, respectively. In (F), curves in green, blue, and red represent La, Rh, and B contributions, respectively. Black dashed line indicates the total phonon DOS. (H) Schematics of many-body effects that create an extended SP-VHS at E_F.

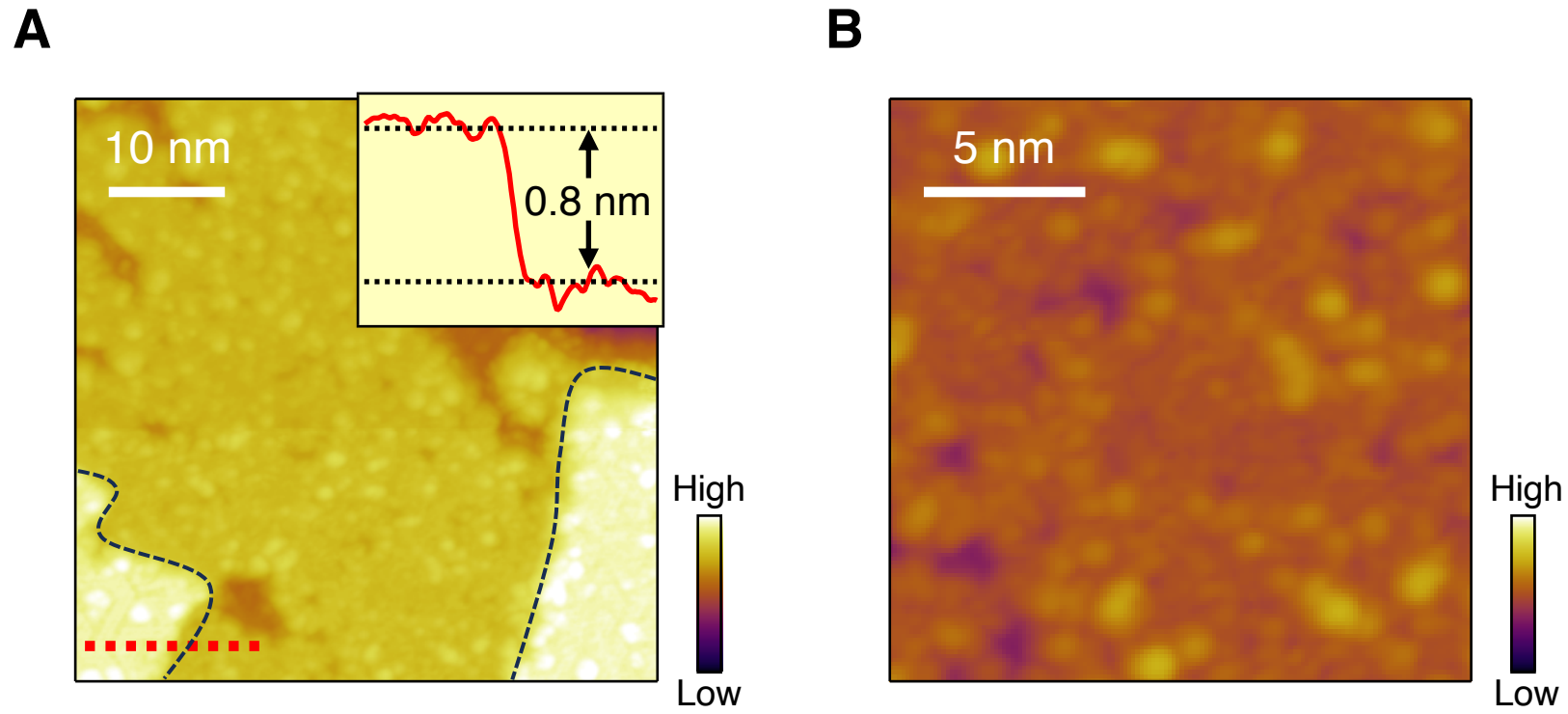

Fig. S7. Another surface observed by STM.

(A) A topographic STM image showing two kinds of surface morphologies separated by steps (black dashed curves). Inset shows a height profile at the red dotted line. (B) A zoomed-in image of the disordered surface. STM setup condition (*V*/*I*): −100 mV/100 pA.

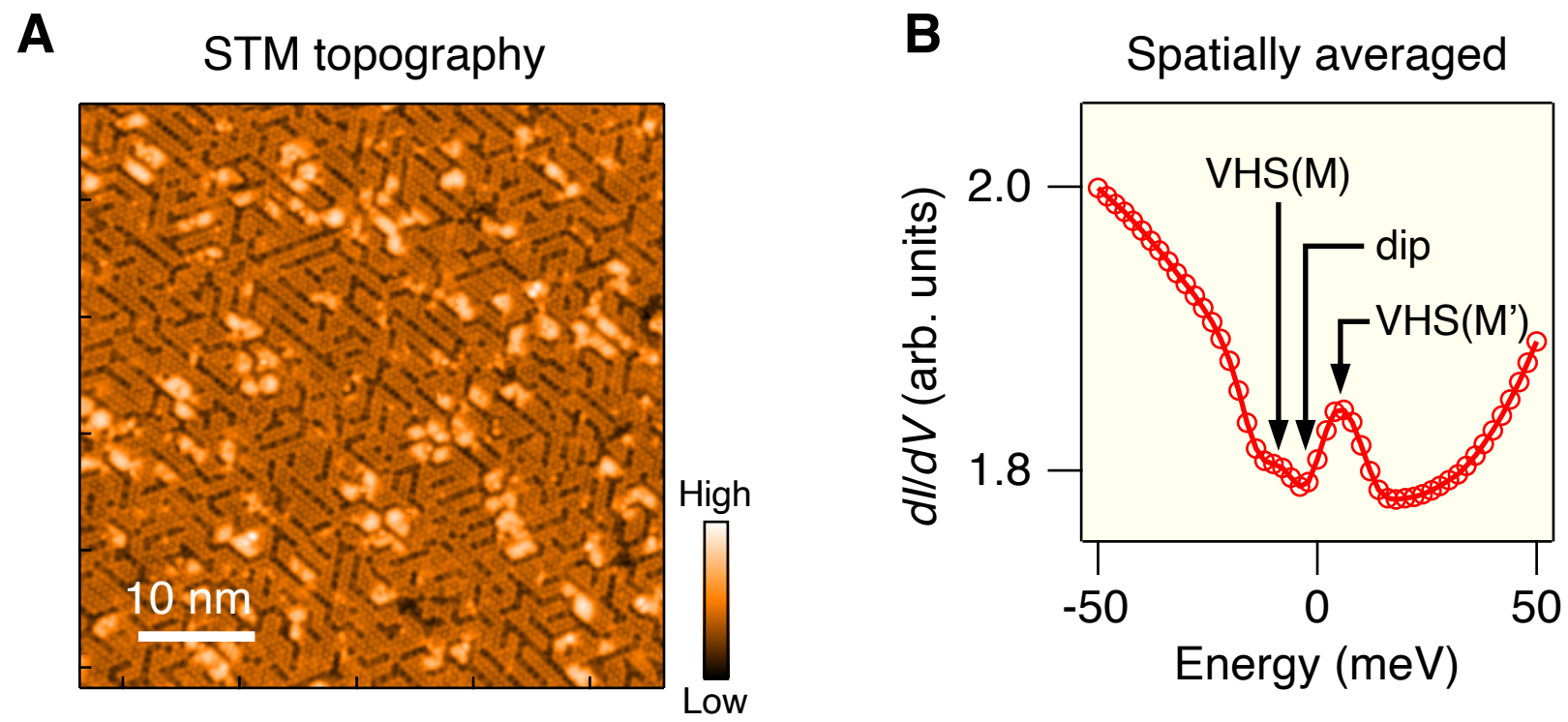

Fig. S8. A d*I*/d*V* spectrum taken in an independent region.

(A,B) Topographic STM image and spatially averaged d*I*/d*V* spectrum with a peak (arrow) measured in a region different from that of Fig. 4. STM setup condition (*V*/*I*): 80 mV/2 nA with a lock-in modulation of 3 mV.

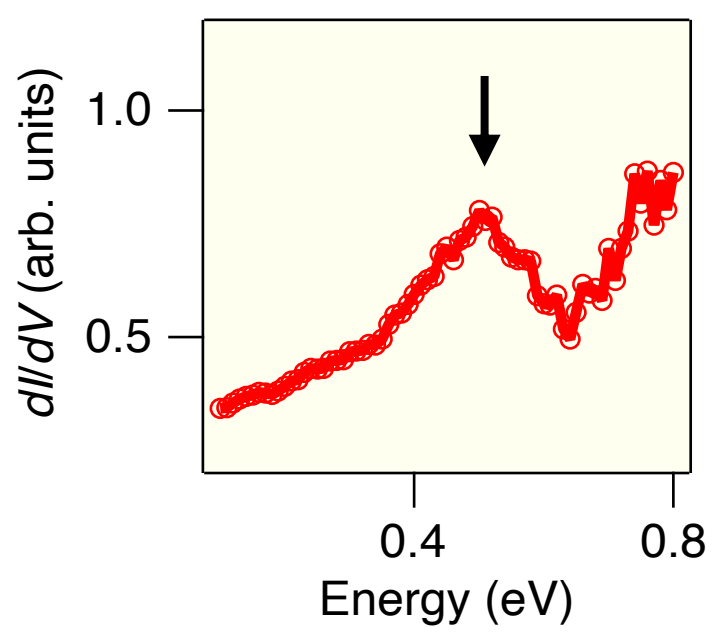

Fig. S9. A peak in d*I*/d*V* observed at a high vias voltage.

A d*I*/d*V* spectrum measured for a wide energy window showing a peak structure (arrow). STM setup condition (*V*/*I*): 1 V/1 nA with a lock-in modulation of 10 mV.

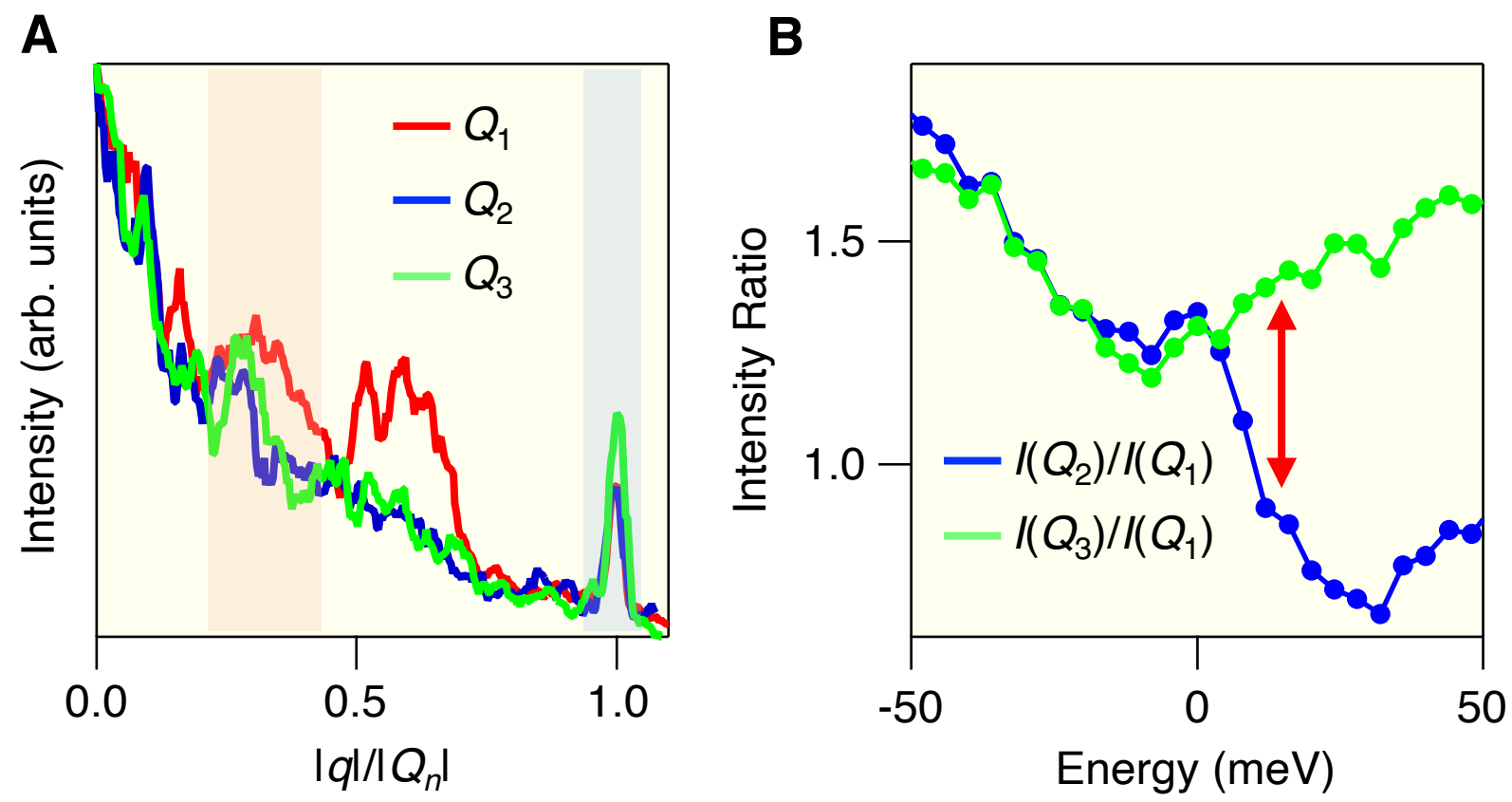

Fig. S10. Supporting data for rotational symmetry breaking.

(A) Line profiles along the Q_1, Q_2, and Q_3 direction (see labels in Fig. 4D). The orange (blue) hatched region indicates the elliptic QPI region (Bragg peak). (B) Energy dependence of the Bragg-peak intensity $I(Q_2)$ (blue) and $I(Q_3)$ (green) normalized by $I(Q_1)$.

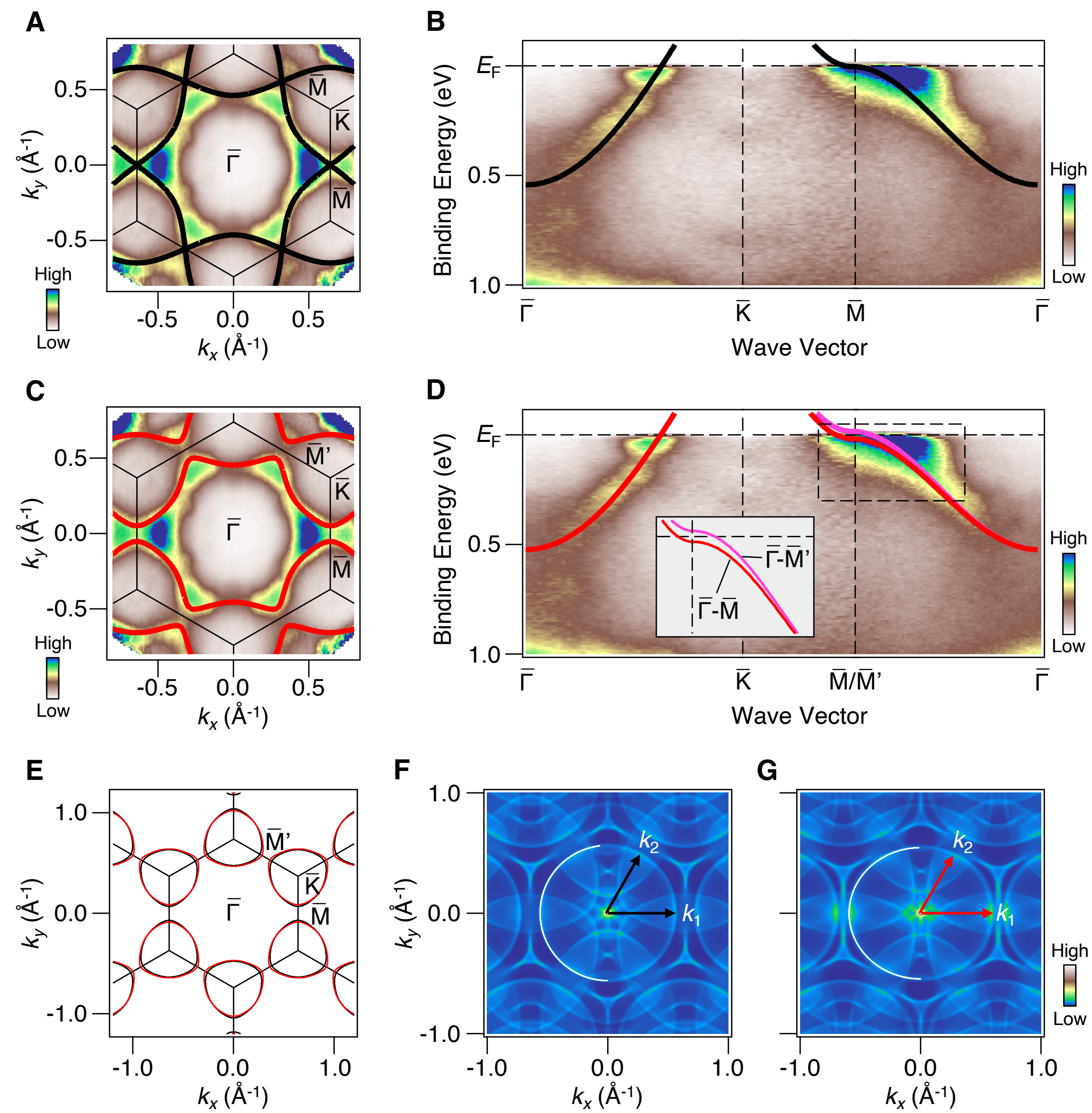

Fig. S11. Tight-binding simulations for observed rotational-symmetry breaking.

(A,B) ARPES-intensity maps at E_F plotted as a function of k_x and k_y and corresponding valence-band structure along the high-symmetry axis, respectively (same as Figs. 2B,C in the main text), compared with tight-binding band calculations. Black curves represent the calculated results with isotropic parameters. (C,D) Same as (A,B) but with anisotropic parameters. Red (magenta) curves in (D) represent the band dispersion along the $\overline{\Gamma\mathrm{KM}\Gamma}$ ($\overline{\Gamma\mathrm{KM'}\Gamma}$) cut. Inset in (D) shows an enlarged view of the region enclosed by the dashed rectangle. (E) Calculated energy contours from a tight-binding model just above the saddle point (+0.03 eV). (F,G) Autocorrelation mapping calculated from the black and red curves in (E), respectively. White half circles are guides for ring pattern and arrows indicate scattering vectors for two $\overline{\Gamma\mathrm{M}}$ directions.

REFERENCES

1. K. S. Novoselov, A. K. Geim, S. V. Morozov, D. Jiang, Y. Zhang, S. V. Dubonos, I. V. Grigorieva, A. A. Firsov, Electric field effect in atomically thin carbon films. *Science* **306**, 666–669 (2004).

2. A. K. Geim, K. Novoselov, The rise of graphene. *Nat. Mater.* **6**, 183–191 (2007).

3. N. M. R. Peres, Colloquium: The transport properties of graphene: An introduction. *Rev. Mod. Phys.* **82**, 2673–2700 (2010).

4. J. González, F. Guinea, M. A. H. Vozmediano, Electron-electron interactions in graphene sheets. *Phys. Rev. B* **63**, 134421 (2001).

5. G. Li, A. Luican, J. M. B. Lopes dos Santos, A. H. Castro Neto, A. Reina, J. Kong, E. Y. Andrei, Observation of Van Hove singularities in twisted graphene layers. *Nat. Phys.* **6**, 109–113 (2010).

6. Y. Cao, V. Fatemi, S. Fang, K. Watanabe, T. Taniguchi, E. Kaxiras, P. Jarillo-Herrero, Unconventional superconductivity in magic-angle graphene superlattices. *Nature* **556**, 43–50 (2018).

7. Y. Cao, V. Fatemi, A. Demir, S. Fang, S. L. Tomarken, J. Y. Luo, J. D. Sanchez-Yamagishi, K. Watanabe, T. Taniguchi, E. Kaxiras, R. C. Ashoori, P. Jarillo-Herrero, Correlated insulator behaviour at half-filling in magic-angle graphene superlattices. *Nature* **556**, 80–84 (2018).

8. C. N. Lau, M. W. Bockrath, K. F. Mak, F. Zhang, Reproducibility in the fabrication and physics of moiré materials. *Nature* **602**, 41–50 (2022).

9. L. Van Hove, The occurrence of singularities in the elastic frequency distribution of a crystal. *Phys. Rev.* **89**, 1189–1193 (1953).

10. N. F. Q. Yuan, H. Isobe, L. Fu, Magic of high-order van Hove singularity. *Nat. Commun.* **10**, 5769 (2019).

11. J. L. McChesney, A. Bostwick, T. Ohta, T. Seyller, K. Horn, J. González, E. Rotenberg, Extended van Hove singularity and superconducting instability in doped graphene. *Phys. Rev. Lett.* **104**, 136803 (2010).

12. B. Valenzuela, M. A. H. Vozmediano, Pomeranchuk instability in doped graphene. *New J. Phys.* **10**, 113009 (2008).

13. C. A. Lamas, D. C. Cabra, N. Grandi, Generalized Pomeranchuk instabilities in graphene. *Phys. Rev. B* **80**, 075108 (2009).

14. R. Nandkishore, L. S. Levitov, A. V. Chubukov, Chiral superconductivity from repulsive interactions in doped graphene. *Nat. Phys.* **8**, 158–163 (2012).

15. W. Li, L. Kong, C. Chen, J. Gou, S. Sheng, W. Zhang, H. Li, L. Chen, P. Cheng, K. Wu, Experimental realization of honeycomb borophene. *Sci. Bull.* **63**, 282–286 (2018).

16. H. C. Ku, G. P. Meisner, F. Acker, D. C. Johnston, Superconducting and magnetic properties of new ternary borides with the $CeCo_3B_2$-type structure. *Solid State Commun.* **35**, 91–96 (1980).

17. H. Barz, New ternary superconductors with silicon. *Mater. Res. Bull.* **15**, 1489–1491 (1980).

18. J. M. Vandenberg, H. Barz, The crystal structure of a new ternary silicide in the system rare-earth-ruthenium-silicon. *Mater. Res. Bull.* **15**, 1493–1498 (1980).

19. Y. Iida, S. Souma, T. Sato, T. Takahashi, M. Yamada, T. Okubo, T. Shiromoto, Y. Inada, R. Settai, Y. Onuki, High-resolution angle-resolved photoemission study of $LaRh_3B_2$. *Physica B* **351**, 271–273 (2004).

20. M. Yamada, Y. Obiraki, T. Okubo, T. Shiromoto, Y. Kida, M. Shiimoto, H. Kohara, T. Yamamoto, D. Honda, A. Galatanu, Y. Haga, T. Takeuchi, K. Sugiyama, R. Settai, K. Kindo, S. K. Dhar, H. Harima, Y. Ōnuki, Magnetic and Fermi surface properties in $PrRh_3B_2$. *J. Physical Soc. Japan* **73**, 2266–2275 (2004).

21. S. Chaudhary, Shama, J. Singh, A. Consiglio, D. Di Sante, R. Thomale, Y. Singh, Role of electronic correlations in the kagome-lattice superconductor $LaRh_3B_2$. *Phys. Rev. B* **107**, 085103 (2023).

22. J. Langen, G. Jackel, W. Schlabitz, M. Veit, D. Wohlleben, Lattice parameter and resistivity anomalies of $CeRh_3B_2$. *Solid State Commun.* **64**, 169–173 (1987).

23. D. Makogon, R. van Gelderen, R. Roldán, C. M. Smith, Spin-density-wave instability in graphene doped near the van Hove singularity. *Phys. Rev. B* **84**, 125404 (2011).

24. R. Nandkishore, G.-W. Chern, A. V. Chubukov, Itinerant half-metal spin-density-wave state on the hexagonal lattice. *Phys. Rev. Lett.* **108**, 227204 (2012).

25. W.-S. Wang, Z.-Z. Li, Y.-Y. Xiang, Q.-H. Wang, Competing electronic orders on kagome lattices at van Hove filling. *Phys. Rev. B* **87**, 115135 (2013).

26. M. L. Kiesel, C. Platt, R. Thomale, Unconventional Fermi surface instabilities in the Kagome Hubbard model. *Phys. Rev. Lett.* **110**, 126405 (2013).

27. I. I. Pomeranchuk, On the stability of a Fermi liquid. *J. Exp. Theor. Phys.* **8**, 361–362 (1959).

28. X. Han, A. P. Schnyder, X. Wu, Enhanced nematicity emerging from higher-order Van Hove singularities. *Phys. Rev. B* **107**, 184504 (2023).

29. Y. Ando, K. Segawa, S. Komiya, A. N. Lavrov, Electrical resistivity anisotropy from self-organized one dimensionality in high-temperature superconductors. *Phys. Rev. Lett.* **88**, 137005 (2002).

30. R. Daou, J. Chang, D. Leboeuf, O. Cyr-Choinière, F. Laliberté, N. Doiron-Leyraud, B. J. Ramshaw, R. Liang, D. A. Bonn, W. N. Hardy, L. Taillefer, Broken rotational symmetry in the pseudogap phase of a high-T_c superconductor. *Nature* **463**, 519–522 (2010).

31. M. J. Lawler, K. Fujita, J. Lee, A. R. Schmidt, Y. Kohsaka, C. K. Kim, H. Eisaki, S. Uchida, J. C. Davis, J. P. Sethna, E.-A. Kim, Intra-unit-cell electronic nematicity of the high-T_c copper-oxide pseudogap states. *Nature* **466**, 347–351 (2010).

32. V. Hinkov, D. Haug, B. Fauqué, P. Bourges, Y. Sidis, A. Ivanov, C. Bernhard, C. T. Lin, B. Keimer, Electronic liquid crystal state in the high-temperature superconductor $YBa_2Cu_3O_{6.45}$. *Science* **319**, 597–600 (2008).

33. R. M. Fernandes, L. H. VanBebber, S. Bhattacharya, P. Chandra, V. Keppens, D. Mandrus, M. A. McGuire, B. C. Sales, A. S. Sefat, J. Schmalian, Effects of nematic fluctuations on the elastic properties of iron arsenide superconductors. *Phys. Rev. Lett.* **105**, 157003 (2010).

34. J.-H. Chu, H.-H. Kuo, J. G. Analytis, I. R. Fisher, Divergent nematic susceptibility in an iron arsenide superconductor. *Science* **337**, 710–712 (2012).

35. L. Nie, K. Sun, W. Ma, D. Song, L. Zheng, Z. Liang, P. Wu, F. Yu, J. Li, M. Shan, D. Zhao, S. Li, B. Kang, Z. Wu, Y. Zhou, K. Liu, Z. Xiang, J. Ying, Z. Wang, T. Wu, X. Chen, Charge-density-wave-driven electronic nematicity in a kagome superconductor. *Nature* **604**, 59–64 (2022).

36. Y. Hu, C. Le, Y. Zhang, Z. Zhao, J. Liu, J. Ma, N. C. Plumb, M. Radovic, H. Chen, A. P. Schnyder, X. Wu, X. Dong, J. Hu, H. Yang, H.-J. Gao, M. Shi, Non-trivial band topology and orbital-selective electronic nematicity in a titanium-based kagome superconductor. *Nat. Phys.* **19**, 1827–1833 (2023).

37. H. Li, S. Cheng, B. R. Ortiz, H. Tan, D. Werhahn, K. Zeng, D. Johrendt, B. Yan, Z. Wang, S. D. Wilson, I. Zeljkovic, Electronic nematicity without charge density waves in titanium-based kagome metal. *Nat. Phys.* **19**, 1591–1598 (2023).

38. Y.-X. Jiang, S. Shao, W. Xia, M. Denner, J. Ingham, M. S. Hossain, Q.-Y. Qiu, X. Zheng, H. Chen, Z.-J. Cheng, X. P. Yang, B. Kim, J.-X. Yin, S. Zhang, M. Litskevich, Q. Zhang, T. Cochran, Y. Peng, G. Chang, Y. Guo, R. Thomale, T. Neupert, M. Hasan, Van Hove annihilation and nematic instability on a kagome lattice. *Nat. Mater.* **23**, 1214–1221 (2024).

39. T. Okubo, M. Yamada, A. Thamizhavel, S. Kirita, Y. Inada, R. Settai, H. Harima, K. Takegahara, A. Galatanu, E. Yamamoto, Y. Ōnuki, Unique Fermi surfaces with quasi-one-dimensional character in $CeRh_3B_2$ and $LaRh_3B_2$. *J. Phys. Condens. Matter* **15**, L721 (2003).

40. S. K. Malik, A. M. Umarji, G. K. Shenoy, A. T. Aldred, D. G. Niarchos, Magnetism and superconductivity in the system $Ce_{1-x}La_xRh_3B_2$. *Phys. Rev. B* **32**, 4742–4745 (1985).

41. G. Henkelman, A. Arnaldsson, H. Jónsson, A fast and robust algorithm for Bader decomposition of charge density. *Comput. Mater. Sci.* **36**, 354–360 (2006).

42. P. Giannozzi, S. Baroni, N. Bonini, M. Calandra, R. Car, C. Cavazzoni, D. Ceresoli, G. L. Chiarotti, M. Cococcioni, I. Dabo, A. Dal Corso, S. de Gironcoli, S. Fabris, G. Fratesi, R. Gebauer, U. Gerstmann, C. Gougoussis, A. Kokalj, M. Lazzeri, L. Martin-Samos, N. Marzari, F. Mauri, R. Mazzarello, S. Paolini, A. Pasquarello, L. Paulatto, C. Sbraccia, S. Scandolo, G. Sclauzero, A. P. Seitsonen, A. Smogunov, P. Umari, R. M. Wentzcovitch, QUANTUM ESPRESSO: A modular and open-source software project for quantum simulations of materials. *J. Phys. Condens. Matter* **21**, 395502 (2009).

43. P. Giannozzi, O. Andreussi, T. Brumme, O. Bunau, M. Buongiorno Nardelli, M. Calandra, R. Car, C. Cavazzoni, D. Ceresoli, M. Cococcioni, N. Colonna, I. Carnimeo, A. Dal Corso, S. de Gironcoli, P. Delugas, R. A. DiStasio Jr, A. Ferretti, A. Floris, G. Fratesi, G. Fugallo, R. Gebauer, U. Gerstmann, F. Giustino, T. Gorni, J. Jia, M. Kawamura, H.-Y. Ko, A. Kokalj, E. Küçükbenli, M. Lazzeri, M. Marsili, N. Marzari, F. Mauri, N. L. Nguyen, H.-V. Nguyen, A. Otero-de-la-Roza, L. Paulatto, S. Poncé, D. Rocca, R. Sabatini, B. Santra, M. Schlipf, A. P. Seitsonen, A. Smogunov, I. Timrov, T. Thonhauser, P. Umari, N. Vast, X. Wu, S. Baroni, Advanced capabilities for materials modelling with Quantum ESPRESSO. *J. Phys. Condens. Matter* **29**, 465901 (2017).

44. J. P. Perdew, K. Burke, M. Ernzerhof, Generalized gradient approximation made simple. *Phys. Rev. Lett.* **77**, 3865–3868 (1996).

45. P. E. Blöchl, Projector augmented-wave method. *Phys. Rev. B* **50**, 17953–17979 (1994).

46. Y. M. Koroteev, G. Bihlmayer, J. E. Gayone, E. V. Chulkov, S. Blügel, P. M. Echenique, P. Hofmann, Strong spin-orbit splitting on Bi surfaces. *Phys. Rev. Lett.* **93**, 046403 (2004).

47. J. González, F. Guinea, M. A. H. Vozmediano, Renormalization group approach to the normal state of copper-oxide superconductors. *Nucl. Phys. B.* **485**, 694–724 (1997).

48. R. Roldán, M. P. López-Sancho, F. Guinea, Effect of electron-electron interaction on the Fermi surface topology of doped graphene. *Phys. Rev. B* **77**, 115410 (2008).

49. J. Yu, Z. Xu, K. Xiao, Y. Yuan, Q. Yin, Z. Hu, C. Gong, Y. Guo, Z. Tu, P. Tang, H. Lei, Q.-K. Xue, W. Li, Evolution of electronic structure in pristine and Rb-reconstructed surfaces of kagome metal RbV_3Sb_5. *Nano Lett.* **22**, 918–925 (2022).

50. A. Berkó, F. Solymosi, Adsorption-induced structural changes of Rh supported by TiO_2(110)-(1×2): An STM study. *J. Catal.* **183**, 91–101 (1999).

51. V. M. Pereira, A. H. Castro Neto, N. M. R. Peres, Tight-binding approach to uniaxial strain in graphene. *Phys. Rev. B* **80**, 045401 (2009).

52. Y.-X. Jiang, J.-X. Yin, M. M. Denner, N. Shumiya, B. R. Ortiz, G. Xu, Z. Guguchia, J. He, M. S. Hossain, X. Liu, J. Ruff, L. Kautzsch, S. S. Zhang, G. Chang, I. Belopolski, Q. Zhang, T. A. Cochran, D. Multer, M. Litskevich, Z.-J. Cheng, X. P. Yang, Z. Wang, R. Thomale, T. Neupert, S. D. Wilson, M. Z. Hasan, Unconventional chiral charge order in kagome superconductor KV_3Sb_5. *Nat. Mater.* **20**, 1353–1357 (2021).

53. S. Reich, J. Maultzsch, C. Thomsen, P. Ordejón, Tight-binding description of graphene. *Phys. Rev. B* **66**, 035412 (2002).
